# Targeting Pyruvate Carboxylase by a Small Molecule Suppresses Breast Cancer Progression

**DOI:** 10.1002/advs.201903483

**Published:** 2020-03-12

**Authors:** Qingxiang Lin, Yuan He, Xue Wang, Yong Zhang, Meichun Hu, Weikai Guo, Yundong He, Tao Zhang, Li Lai, Zhenliang Sun, Zhengfang Yi, Mingyao Liu, Yihua Chen

**Affiliations:** ^1^ East China Normal University and Shanghai Fengxian District Central Hospital Joint Center for Translational Medicine Shanghai Key Laboratory of Regulatory Biology Institute of Biomedical Sciences and School of Life Sciences East China Normal University Shanghai 200241 P. R. China; ^2^ Joint Center for Translational Medicine Southern Medical University Affiliated Fengxian Hospital Shanghai 201499 P. R. China

**Keywords:** breast cancer, cancer metabolism, pyruvate carboxylase, small molecule ZY‐444, Wnt/β‐catenin/Snail pathway

## Abstract

Rapid metabolism differentiates cancer cells from normal cells and relies on anaplerotic pathways. However, the mechanisms of anaplerosis‐associated enzymes are rarely understood. The lack of potent and selective antimetabolism drugs restrains further clinical investigations. A small molecule ZY‐444 ((*N* ^4^‐((5‐(4‐(benzyloxy)phenyl)‐2‐thiophenyl)methyl)‐*N* ^2^‐isobutyl‐2,4‐pyrimidinediamine) is discovered to inhibit cancer cell proliferation specifically, having potent efficacies against tumor growth, metastasis, and recurrence. ZY‐444 binds to cellular pyruvate carboxylase (PC), a key anaplerotic enzyme of the tricarboxylic acid cycle, and inactivates its catalytic activity. PC inhibition suppresses breast cancer growth and metastasis through inhibiting the Wnt/β‐catenin/Snail signaling pathway. Lower PC expression in patient tumors is correlated with significant survival benefits. Comparative profiles of PC expression in cancer versus normal tissues implicate the tumor selectivity of ZY‐444. Overall, ZY‐444 holds promise therapeutically as an anti‐cancer metabolism agent.

## Graphical abstract

A specific metabolic inhibitor (named ZY‐444) of pyruvate carboxylase (PC), the key anaplerosis enzyme, is discovered to exhibit cancer selectivity as well as great therapeutic efficacy against breast cancer progression. This study also demonstrates the mechanistic roles of PC in breast cancer progression and identifies the crosstalk between the canonical Wnt pathway and cancer anaplerosis.

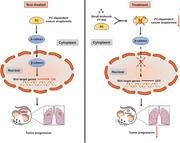

## 1 Introduction

Oncogenetic reprogramming of cancer metabolism favors the Warburg effect, a shift to accelerated glycolysis under aerobic environments,^[^ ^1^ ^]^ to meet the increased anabolic and energetic demands for proliferation and migration.^[^ ^2^ ^]^ However, enhanced glycolysis alone is insufficient to provide all essential metabolic intermediates for tumor growth and metastasis.^[^ ^3^ ^]^ To facilitate cell proliferation, tumor cells frequently adapt by elevated anaplerosis,^[^ ^4^ ^]^ which replenishes the biosynthetic precursor molecules to the tricarboxylic acid cycle (TCA), such as oxaloacetate and glutamine,^[^ ^3^, ^5^ ^]^ as well as essential intermediates of the mitochondrial electron chain.^[^ ^6^ ^]^ Along with the high glycolytic flux, a high anaplerotic flux could indicate excessive cancer proliferation.^[^ ^7^ ^]^

Two enzymes that play indispensable roles in anaplerosis of TCA intermediates are glutaminase and pyruvate carboxylase (PC).^[^ ^8^ ^]^ PC, which is present in the mitochondria, oxidizes pyruvate into oxaloacetate. The role of PC has been implicated in cancer progression. Compared with non‐cancerous tissues, PC expression is greatly enhanced in patients with non‐small cell lung cancer (NSCLC) and invasive breast cancer (BCA).^[^ ^9^ ^]^ In early stages of NSCLC, growth inhibition by PC knockdown is accompanied by disrupted TCA activity and biosynthesis.^[^ ^10^ ^]^ In particular, BCA accounts for the highest frequency of PC amplification according to The Cancer Genome Atlas (TCGA). In vitro, PC supports proliferation and invasion of BCA cells.^[^ ^11^ ^]^ Importantly, BCA lung metastases develop a significantly higher PC‐catalyzed generation of oxaloacetate, along with an increased intracellular pyruvate level as compared to primary tumors.^[^ ^9^, ^12^ ^]^ Researchers also unveiled a potential metabolic vulnerability of glutamine independent cancer cells through inhibiting PC activity.^[^ ^6^, ^13^ ^]^ Thus, PC could be a promising therapeutic target for cancer progression.

PC is regulated at metabolic and transcriptional levels. Physiologically, glutamate and α‐ketoglutarate inhibit its expression. Several transcription factors regulate PC expression, including Snail.^[^ ^14^ ^]^ However, the core signaling pathway by which PC modulates cancer metabolism is not well understood.^[^ ^15^ ^]^ In diabetes, the canonical Wnt signaling pathway is heavily implicated in deregulation of metabolic homeostasis. Yet, the critical role of Wnt signaling in cancer metabolism remains under‐investigated. Besides, mitochondria‐mediated retrograde signaling modulates Wnt signaling, and impaired mitochondrial ATP production downregulates Wnt signaling via ER stress induction.^[^ ^16^ ^]^ Moreover, activation of the Wnt signaling pathway is also a glycolytic switch to enhance glucose consumption and lactate production; they also reported an induction of PC that was dependent on Snail and TCF‐4. Lee and co‐workers didn't detect direct Snail binding to the PC promoter, suggesting an indirect mechanism of transcriptional regulation.^[^ ^14^ ^]^ However, it has not been elucidated whether PC could in turn regulate the canonical Wnt signaling pathway.

Treatment targeting tumor metabolism holds promise to improve prognosis.^[^ ^17^ ^]^ However, poor tumor selectivity precludes many preclinical anti‐metabolic agents from clinical use.^[^ ^18^ ^]^ Notably, Kumashiro et al. showed low toxicity to non‐cancerous tissues by targeting PC in vivo.^[^ ^19^ ^]^ Nevertheless, the lack of effective and selective inhibitors against PC hinders clinical investigation.^[^ ^20^ ^]^

To develop potent metabolic inhibitors with tumor selectivity, a series of compounds containing aryl‐heteroaromatic and pyrimidinediamine scaffolds were designed, synthesized and optimized through comparing their inhibitory effects upon normal versus multiple cancer cell lines. Interestingly, most synthesized derivatives were able to regulate mitochondrial respiration as determined by Seahorse Mito Stress assay, partially because they share a similar chemical scaffold of aryl‐heteroaromatic as well as pyrimidinediamine groups. Compound (*N* ^4^‐((5‐(4‐(benzyloxy)phenyl)‐2‐thiophenyl)methyl)‐*N* ^2^‐isobutyl‐2,4‐pyrimidine‐diamine (molecular weight 444 kDa, named ZY‐444) was identified among the investigated derivatives by our screening workflow. ZY‐444 manifested potent efficacy against cancer proliferation and invasion but much less toxicity to normal cells. ZY‐444 strongly and selectively decreased basal respiration and ATP production in BCA cells compared to its effects in normal epithelial cells. It bound to PC and inhibited its activity. Gain‐ and loss of function assays suggested that PC was not only indispensable for BCA growth and metastasis but also required for the activity of ZY‐444 on cancer metabolism. Furthermore, we observed the role of PC in modulation of the Wnt/β‐catenin/Snail pathway. Notably, we found that higher PC expression was correlated with poor prognostic outcomes in multiple cancer types whereas corresponding normal tissues express lower PC. Therefore, ZY‐444 holds promise as a potent and selective PC inhibitor in cancer therapies.

## 2 Results

### 2.1 Screening for Potent Anti‐Cancer Metabolic Compounds

Targeting cancer metabolism has emerged as an effective strategy for the development of selective antitumor drugs.^[^ ^17^, ^21^ ^]^ Therapeutically, the availability of FDA‐approved anti‐metabolic agents is limited owing to the challenge of dose‐limiting toxicity, and their inadequate potency in preclinical studies.^[^ ^17^, ^22^ ^]^ To address this problem, we established a drug screening workflow (Figure S1A, Supporting Information). Initially, a library of ≈500 compounds with diverse structures was screened in breast, lung, and colon cancer cell lines. Several lead hits including WB‐339B were characterized based on their anti‐cancer activities whereas most were poorly selective for cancer cells. We hypothesized that compound tumor selectivity could partly result from differential metabolism in tumor cells versus normal cells. Currently, some metabolism inhibitors are undergoing preclinical and clinical investigations.^[^ ^3^, ^23^ ^]^ For example, IACS‐010759 robustly induces energy depletion and aspartate biosynthesis reduction, leading to strong apoptosis in preclinical cancer models dependent on oxidative phosphorylation.^[^ ^3b^ ^]^ It also displays differential sensitivity between normal and cancer cells. We noticed that WB‐339B possesses similar chemical structures “aryl‐heteroaromatic groups” (highlighted in blue, Table S1 and Figure S1B, Supporting Information) to IACS‐010759 and other metabolism regulators (YC‐1,^[^ ^23b^ ^]^ AGI‐6780,^[^ ^24^ ^]^ and BAY 87–2243^[^ ^23a^ ^]^). This structural similarity prompted us to test whether WB‐339B could also inhibit cell metabolism. The Seahorse Mito Stress test enables measuring the oxygen consumption rate (OCR) and calculating key parameters of mitochondrial functions. As expected, WB‐339B strongly inhibited mitochondrial respiration of MDA‐MB‐231 cells, indicating an inhibitory potential against cancer metabolism (Figure S1C,D, Supporting Information). Therefore, more than 50 derivatives of WB‐339B were synthesized and optimized to determine the relationship between structure and anti‐cancer activities.^[^ ^25^ ^]^ Some derivatives, such as ZY‐444 and ZY‐459, strongly inhibited mitochondrial respiration, reflecting strong antimetabolism properties at 10 and 20 × 10^−6^ m (Figure S1C,D, Supporting Information). Interestingly, compounds (WK‐325, WB‐464, and WK‐400B) structurally lacking aryl‐heteroaromatic groups did not suppress mitochondrial respiration (Figure S1E,F, Supporting Information). These results together suggested that structural incorporation of aryl‐heteroaromatic groups could potentially impair hyperactive mitochondrial functions.

**Figure 1:**
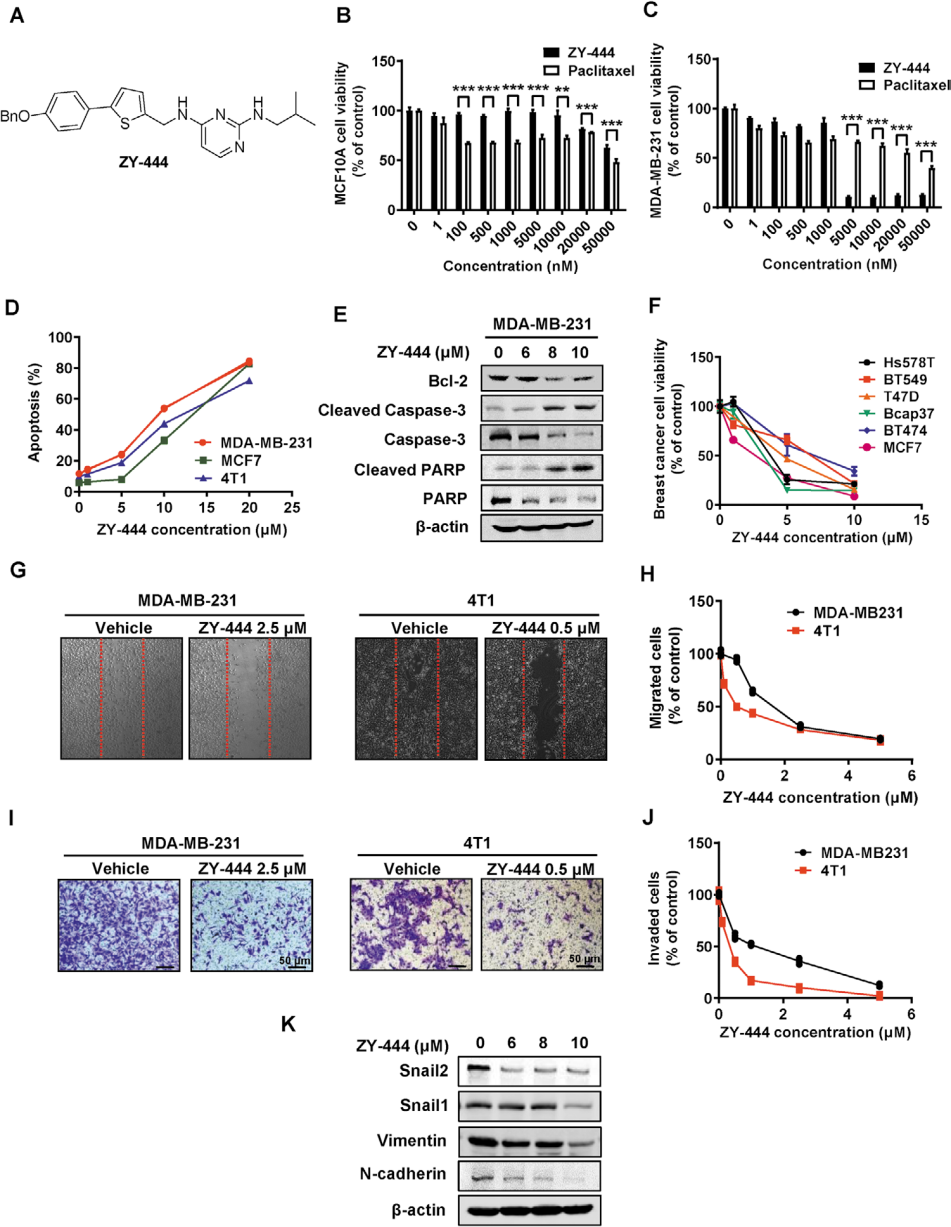
A) ZY‐444 selectively inhibited BCA proliferation, migration, and invasion. The chemical structure of ZY‐444. B,C) Effects of paclitaxel and ZY‐444 on cell viability in normal epithelial cells (B, MCF10A) and BCA cells (C, MDA‐MB‐231). D) Effects of ZY‐444 on cell apoptosis and E) the expression of apoptosis markers. F) Effects of ZY‐444 on cell viability in several BCA cell lines. G–J) Effects of ZY‐444 upon cell migration and invasion of BCA cell lines (MDA‐MB‐231 and 4T1) in G,H) the wound healing assay and I,J) transwell invasion assay. Representative images and quantification of cell counts normalized by controls are shown. K) Effects of ZY‐444 on mesenchymal marker expression in MDA‐MB‐231 cells. Data shown are mean ± s.d. *p* < 0.01, **; *p* < 0.001, ***.

The structural optimization resulted in greater tumor selectivity. Four WB‐339B derivatives were selected due to their potencies on many cancer cell lines. ZY‐444 was chosen for further investigation because it had the highest tumor selectivity (**Figure** **1**A–C) and impairment of cancer mitochondrial respiration (Figure S1C,D, Supporting Information). Considering that BCA cells were more sensitive to ZY‐444 among the investigated cancer cell lines (Figure 1C; Figure S2A,B, Supporting Information), we initially determined to investigate ZY‐444 in BCA.

### 2.2 ZY‐444 Selectively Suppressed BCA Cell Proliferation with Low Cytotoxicity in Normal Cells

BCA is the most common malignancy and a leading cause of female cancer death. Paclitaxel is a first‐line therapeutic agent for BCA.^[^ ^26^ ^]^ To compare the tumor selectivity of paclitaxel and ZY‐444, the MTS assay was employed in normal breast (MCF10A) versus BCA cells. In MCF10A cells, 100 × 10^−9^ m paclitaxel significantly repressed cell proliferation whereas cells were insensitive to even 10 × 10^−6^ m ZY‐444 (Figure 1B), suggesting ZY‐444 was less toxic to normal cells than paclitaxel in vitro. In BCA cells, paclitaxel could not achieve 50% inhibitory effects even at 20 × 10^−6^ m. In contrast, ZY‐444 inhibited cancer cell viability by 90% at 5 × 10^−6^ m (Figure 1C). The percentages of apoptotic cells sharply arose at 5 and 10 × 10^−6^ m ZY‐444 (Figure 1D; Figure S2C, Supporting Information). Furthermore, we detected increases in apoptotic biomarkers (cleaved caspase‐3 and cleaved PARP) and decreased expression of anti‐apoptosis biomarkers Bcl‐2 with ZY‐444 treatment (Figure 1E). Importantly, a significant induction of apoptosis was observed in BCA cells treated with ZY‐444 whereas apoptosis in MCF10A cells was not induced (Figure S2C,D, Supporting Information). Additionally, ZY‐444 was effective against other BCA cell lines (Figure 1F). Thus, ZY‐444 exhibited a higher selectivity of toxicity to BCA cells compared with paclitaxel in vitro.

### 2.3 ZY‐444 Showed Anti‐Migration and Anti‐Invasion Effects In Vitro

Principally, BCA deaths are caused by cancer metastasis.^[^ ^27^ ^]^ Epithelial‐mesenchymal transition (EMT) is a developmental process that converts epithelial cells into mesenchymal and migratory phenotypes, which has been heavily implicated in cancer metastasis.^[^ ^28^ ^]^ Several factors regulate EMT, including Snail, Slug (Snail2), N‐cadherin, and Vimentin.^[^ ^29^ ^]^ To investigate the effects of ZY‐444 on migration and invasion, we conducted wound healing assays and invasion assays. The numbers of the migrating or invasive cells were significantly reduced with ZY‐444 treatment in both cell lines (Figure 1G–J). Consistently, the expression of mesenchymal biomarkers was downregulated in the ZY‐444 group (Figure 1K). Collectively, ZY‐444 could suppress BCA migration and invasion in vitro.

### 2.4 ZY‐444 Blocked Spontaneous Lung Metastases

**Figure 2:**
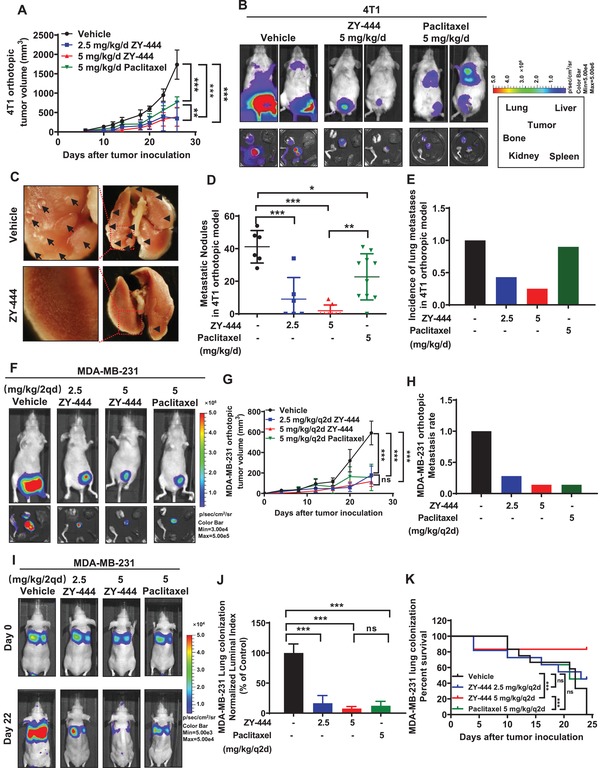
ZY‐444 suppressed BCA growth, metastasis, and lung colonization in vivo. A) ZY‐444 reduced primary tumor growth significantly in the orthotopic 4T1 model (*n* = 6–10). B) Representative bioluminescent images of treated mice and dissected tumors and organs in the 4T1 mouse model. The black rectangle shows the location of dissected tumors and indicated organs in a dish. C) Representative images of lungs in control and 5 mg kg^−1^ ZY‐444 groups. D) Quantification of the numbers of nodules for each treatment. E) Incidence of lung metastases in the 4T1 model. F) Representative bioluminescent images of treated mice as well as resected tumors and organs (same arrangement of organs as in (B) in the MDA‐MB‐231 model. G) ZY‐444 reduced primary tumor growth significantly in an orthotopic MDA‐MB‐231 model (*n* = 7). H) Incidence of organ metastasis (lungs and bones) monitored by ex vivo bioluminescent detection in the MDA‐MB‐231 model. I) The representative bioluminescent images of treated nude mice in the experimental metastasis model. J) The luminal bioluminescent indexes in the lungs of each group were quantified and normalized by an average value of controls. K) The survival rate of each group in the experimental metastasis model was recorded after the tail vein inoculation of MDA‐MB‐231 cells. Data shown are mean ± s.d. *p* < 0.05, *; *p* < 0.01, **; *p* < 0.001, ***; ns, not significant.

To evaluate its therapeutic effects, we next conducted four BCA growth and metastasis models. In the mouse orthotopic BCA model, which mimics the tumor microenvironment, 4T1 cells metastasize to organs, causing metastasis‐associated death.^[^ ^27^ ^]^ In our study, ZY‐444 or paclitaxel treatment reduced the 4T1 tumor burden significantly (**Figure** **2**A). Notably, the mean tumor volume in the ZY‐444 group was significantly lower than the paclitaxel group. Subsequently, we examined tumor metastasis to distant organs using bioluminescent imaging. Interestingly, the photon flux results indicated that 5 mg kg^−1^ ZY‐444 almost entirely blocked signaling from all the distant metastatic sites (Figure 2B). The lungs, being organs with the most visually obvious metastases, were isolated, and the tumor nodules per mouse lungs were counted (Figure 2C). The quantification affirmed that the numbers of tumor nodules in the lungs were reduced greatly in ZY‐444 and paclitaxel groups. Intriguingly, 5 mg kg^−1^ ZY‐444 treatment significantly lowered lung metastatic nodules compared with the paclitaxel group (Figure 2D), with a 25% incidence of visualized lung metastases (2 out of 8 mice, Figure 2E). Hematoxylin–eosin staining of the lung sections verified this result (Figure S3A, Supporting Information). Furthermore, body weight measurements demonstrated no evident weight loss over the course of the treatment (Figure S3B, Supporting Information). In summary, ZY‐444 treatment inhibited tumor growth and metastasis in a spontaneous mouse model, with a higher potency than paclitaxel.

### 2.5 ZY‐444 Inhibited Orthotopic BCA Initiation, Growth, and Metastasis In Vivo

To evaluate the effects of ZY‐444 on tumor initiation and subsequent tumor progression, we employed an MDA‐MB‐231 orthotopic mouse model. 26 days after implantation and dosing, dissected organs and tumors were imaged to detect metastasis. In controls, all the mice had strong bioluminescent signal in tumors and distant organs (Figure 2F). In contrast, ZY‐444 or paclitaxel intensively reduced the bioluminescent signals of primary tumors and the incidence of metastatic signals, without significant body weight loss (Figure 2G; Figure S3C,D, Supporting Information). The overall incidence of distant metastasis in the 5 mg kg^−1^ ZY‐444 group was as same as the paclitaxel group (14%) (Figure 2H). In primary tumors, the expression of apoptotic and mesenchymal biomarkers in ZY‐444‐treated tumors was respectively higher and lower than that in either vehicle‐ or paclitaxel treated tumors (Figure S3E, Supporting Information). Thus, ZY‐444 could inhibit BCA tumor formation and metastasis, and the efficacy was comparable to paclitaxel in vivo.

### 2.6 ZY‐444 Decreased Human BCA Lung Colonization and Prolonged Mouse Survival

To understand the effects of ZY‐444 on the circulation of metastatic cells and tumor colonization on distant organs, the tail vein injection experimental metastasis mouse model was utilized. MDA‐MD‐231‐luc cells were injected into the circulation of female nude mice, followed by drug administration. Luciferase‐expressing tumor cells were detected in the lungs 6 h after cell inoculation (Figure 2I). All control mice were dead on the 24th day, which was generally caused by excessive lung colonization of the cancer cells. On the 22nd day, the photon flux data indicated that the lung metastases in the treatment groups were dramatically reduced (Figure 2I,J). The luminal index of ZY‐444 was marginally lower than that of paclitaxel without statistical significance (Figure 2J). However, survival analysis showed that mice receiving 5 mg kg^−1^ ZY‐444 lived significantly longer than the paclitaxel group (Figure 2K). The average body weight had no significant change over the treatment (Figure S3F, Supporting Information). Taken together, ZY‐444 significantly prolonged the median mouse survival in the experimental metastasis model, with a higher survival rate than paclitaxel in vivo.

### 2.7 ZY‐444 Retarded Local Recurrence and Distant Metastasis After Tumor Excision In Vivo

Local surgeries such as lumpectomy and mastectomy are frequently applied as BCA treatments.^[^ ^30^ ^]^ The incidence of local recurrence and distant metastasis post‐surgery reflects cancer malignancy. We next investigated whether ZY‐444 adjuvant treatment could lower the incidence of local recurrence and distant metastasis, and prolong survival after resection of the initial primary tumors. 4T1 tumor recurrence could be detected using bioluminescent imaging after tumor removal and treatment (Figure S3G, Supporting Information).^[^ ^31^ ^]^ As shown in Figure S3H,I (Supporting Information), control mice showed 80% detectable local recurrence and 40% distant metastasis. All treatments decreased recurrence and distant metastasis. In comparison, the incidence of recurrence in 5 mg kg^−1^ ZY‐444 group was lower than that in paclitaxel cohort. The Kaplan–Meier curves showed that 2.5 mg kg^−1^ qd^−1^ ZY‐444 significantly prolonged survival whereas paclitaxel treatment had no significant improvement in survival (Figure S3J, Supporting Information). Thus, ZY‐444 could suppress local recurrence and distant metastasis, and prolonged survival in an adjuvant mouse model.

### 2.8 ZY‐444 Directly Bound to PC In Vitro

**Figure 3:**
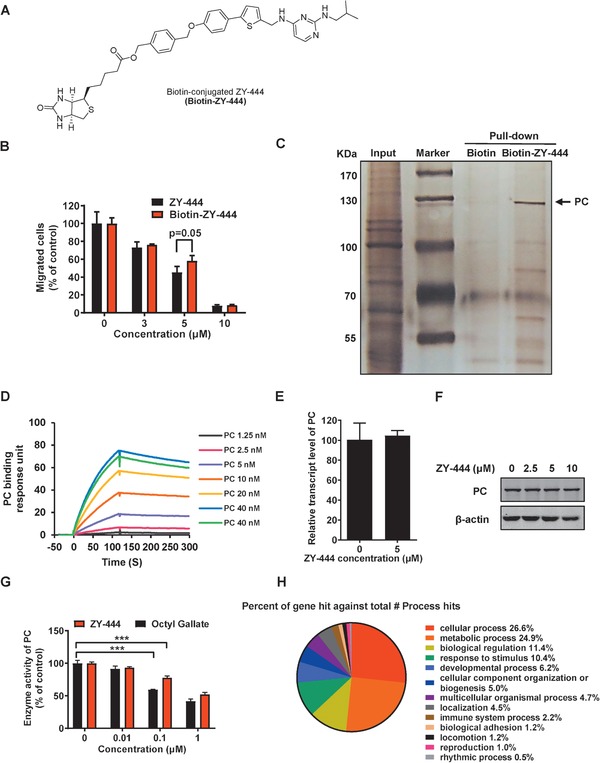
ZY‐444 directly bound to PC and decreased the enzymatic activity of PC. A) The chemical structure of biotin‐conjugated ZY‐444 (Biotin‐ZY‐444). B) The comparative effects of ZY‐444 and biotin‐ZY‐444 on cell migration. C) MDA‐MB‐231 cell lysates were incubated with biotin or biotin‐ZY‐444 overnight. Streptavidin‐conjugated agarose beads were used to precipitate their binding proteins, which were separated by gel electrophoresis and visualized by silver staining. The arrowhead indicates a specific band in the gel, which was identified as PC through mass spectrum analysis. D) The biotin terminal end of biotin‐ZY‐444 was immobilized on the sensor chip. It interacted with the indicated concentrations of purified PC. Strong drug–protein interaction resulted in a series of binding response curves. E,F) PC expression after ZY‐444 exposure for 24 h at E) the mRNA and F) protein levels in MDA‐MB‐231 cells. G) The effects of Octyl gallate and ZY‐444 on PC enzymatic activity. H) A microarray gene expression profiling analysis was performed using MDA‐MB‐231 cells treated with vehicle versus ZY‐444. PANTHER classification analysis provided the percentages of gene hits against total biological hits by ZY‐444. Data shown are mean ± s.d. *p* < 0.001, ***.

The results on mitochondrial respiration encouraged us to explore the binding proteins of ZY‐444 responsible for regulating cancer metabolism. Synthetic derivatives with chemical probes are frequently used to pulldown potential cellular binding targets of the drug.^[^ ^32^ ^]^ Therefore, based on the synthetic route of ZY‐444 (Figure S4A, Supporting Information), we synthesized biotin‐tagged ZY‐444 (Biotin‐ZY‐444) (**Figure** **3**A; Figure S4B, Supporting Information) that showed similar biological activity to ZY‐444 (Figure 3B; Figure S4C,D, Supporting Information).

To identify ZY‐444 interaction proteins, we conducted a pull‐down assay followed by mass spectrometry using biotin‐ZY‐444. In contrast to the biotin lane, one band in the biotin‐ZY‐444 lane, whose molecular weight was around 130 kDa, was consistently precipitated by biotin‐ZY‐444 (Figure 3C). Based on mass spectrometry analyses of the precipitated protein bound to biotin‐ZY‐444, PC was identified with high‐ranking scores (Table S2, Supporting Information). To validate the precipitated protein, the precipitated samples were blotted with an antibody against PC, which supported the notion that the protein precipitated by biotin‐ZY‐444 was PC (Figure S4E, Supporting Information). To substantiate the idea that ZY‐444 directly binds to PC, Surface Plasmon Resonance (SPR) analysis was conducted using biotin‐ZY‐444 for conjugation and various concentrations of PC. Here, the biotinylated terminal was immobilized on the sensor chip. Increasing concentrations of PC enhanced the binding to ZY‐444 (Figure 3D). The binding parameters are shown in Table S3 (Supporting Information), which demonstrated a high affinity between ZY‐444 and PC.

### 2.9 ZY‐444 Downregulated the Enzymatic Activity of PC without Influencing Its Expression

The binding results above urged us to explore the manner that ZY‐444 influenced PC. ZY‐444 did not alter the expression of PC in MDA‐MB‐231 cells at the mRNA and protein levels (Figure 3E,F). Since PC is a metabolic enzyme catalyzing the production of oxaloacetate from pyruvate, we next tested whether ZY‐444 influenced PC enzymatic activity. Generally, PC enzymatic activity is measured by the oxidized rate of NADH followed by multiple metabolic steps. The addition of ZY‐444 concentration dependently inhibited PC activity, similar to *n*‐Octyl gallate, a known inhibitor of PC (Figure 3G).^[^ ^33^ ^]^ To sum up, ZY‐444 is an inhibitor of PC by binding to and inhibiting its enzymatic activity without influencing PC expression.

### 2.10 ZY‐444 Primarily Affected Cancer Metabolism at a Genome‐Wide Level

To support the idea that ZY‐444 influences cancer metabolism, a genome‐wide expression microarray analysis was carried out (Figure 3H). Based on PANTHER classification analysis,^[^ ^34^ ^]^ ZY‐444 significantly altered of metabolic processes (100 genes, 24.9% out of total hits of biological processes), which was ranked second to cellular processes (107 genes, 26.6% out of total hits of biological processes) (Figure 3H; Figure S5A,B, Supporting Information). Additionally, 27.3% (70 genes) of significantly changed genes by ZY‐444 were associated with catalytic activity, which was the second highest molecular function (Figure S5C–E, Supporting Information). The results of our microarray assay substantiate that ZY‐444 principally modulates cancer metabolism in a genome‐wide pattern, and that the metabolically relevant protein PC can be a direct target of ZY‐444.

### 2.11 Pharmacological and Genetic Interference with PC Suppressed the Wnt/β‐Catenin/Snail Pathway

**Figure 4:**
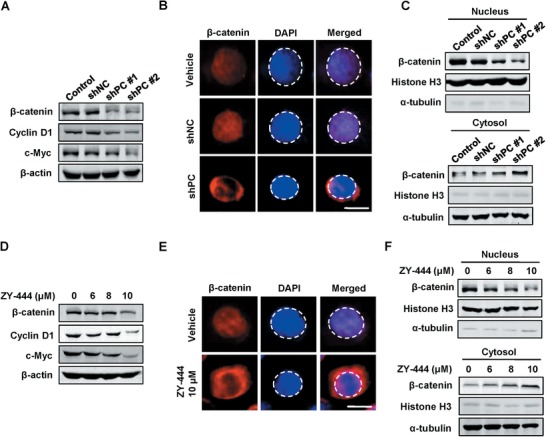
Both the deletion of PC and ZY‐444 treatment inhibited the Wnt signaling pathway in MDA‐MB‐231 cells. A) The expression of key downstream proteins of the Wnt signaling pathway in control cells, shNC, shPC #1, and shPC #2 cells. B) The representative immunofluorescent images for control, shNC, and shPC MDA‐MB‐231 cells for β‐catenin (red) and DAPI (blue) signals. The DAPI signal was circled using dotted lines. Scale bar = 5 µm. C) The protein expression of β‐catenin and Histone H3 in nuclear and cytosolic fractions of control, shNC, and shPC MDA‐MB‐231 cells. D) The expression of downstream proteins of the Wnt signaling pathway in MDA‐MB‐231 cells under ZY‐444 treatment. E) Representative images of stained MDA‐MB‐231 cells treated with vehicle or ZY‐444. The red and blue staining represent β‐catenin and nucleus, respectively. The nucleus was circled using a dotted line. Scale bar = 5 µm. F) Effects of ZY‐444 on β‐catenin distribution between nuclear and cytosolic components in MDA‐MB‐231 cells.

To date, the molecular mechanisms by which PC influences tumor growth and metastasis remain under‐studied. As a critical enzyme of anaplerosis, PC expression was increased downstream of the Wnt/β‐catenin/Snail signaling pathway, and Wnt signaling increased glucose consumption and lactate production.^[^ ^14^ ^]^ For decades, Wnt/β‐catenin signaling has been regarded as a key regulator of cancer progression. Wnt signaling also regulates whole‐body metabolism by altering non‐transformed cell types and tissues.^[^ ^15^, ^35^ ^]^ During osteoblast differentiation, Wnt/mTORC1 signaling regulates the key catabolic enzyme at the first step of glutamine‐dependent anaplerosis,^[^ ^36^ ^]^ implicating a significant role of Wnt signaling in anaplerosis. In recent years, emerging evidence highlights the roles of Wnt in regulating and reprogramming tumor cell metabolism.^[^ ^37^ ^]^ The linear correlation of PC and β‐catenin expression (CTNNB1) in the TCGA co‐expression database suggests the crosstalk of PC and Wnt signaling in multiple cancer types (Figure S6A, Supporting Information).^[^ ^38^ ^]^ Given the effects of the Wnt pathway on cancer metabolism, we hypothesized that PC and known upstream Wnt/β‐catenin/Snail signaling could be mutually modulated in anaplerosis. PC silencing reduced the total expression of key Wnt signaling pathway targets in MDA‐MB‐231 cells, including β‐catenin, c‐Myc and Cyclin D1 (**Figure** **4**A; Figure S6B, Supporting Information), suggesting PC in turn contributed to the activity of Wnt/β‐catenin signaling. As transcriptional function of β‐catenin requires its nuclear translocation, we next examined β‐catenin distribution between subcellular cytoplasmic and nuclear fractions. PC knockdown resulted in a significant decrease of β‐catenin in the nucleus as well as an apparent upregulation of cytosolic β‐catenin levels (Figure 4B,C). Similarly, ZY‐444 lowered the total amount of β‐catenin and invasiveness‐associated biomarkers (Figure 4D; Figure S6B,C, Supporting Information). Consistent with our knockdown results, ZY‐444 prevented the translocation of β‐catenin into the nucleus (Figure 4E,F). Similarly, ZY‐444 lowered the expression of total β‐catenin/Snail and downstream Wnt signaling in the MDA‐MB‐231 orthotopic tumors (Figure S3E, Supporting Information). These results together indicated that inhibition of PC expression impaired Wnt signaling through inducing the cytosolic accumulation of β‐catenin.

### 2.12 PC Knockdown Reduced BCA Growth In Vitro and In Vivo

**Figure 5:**
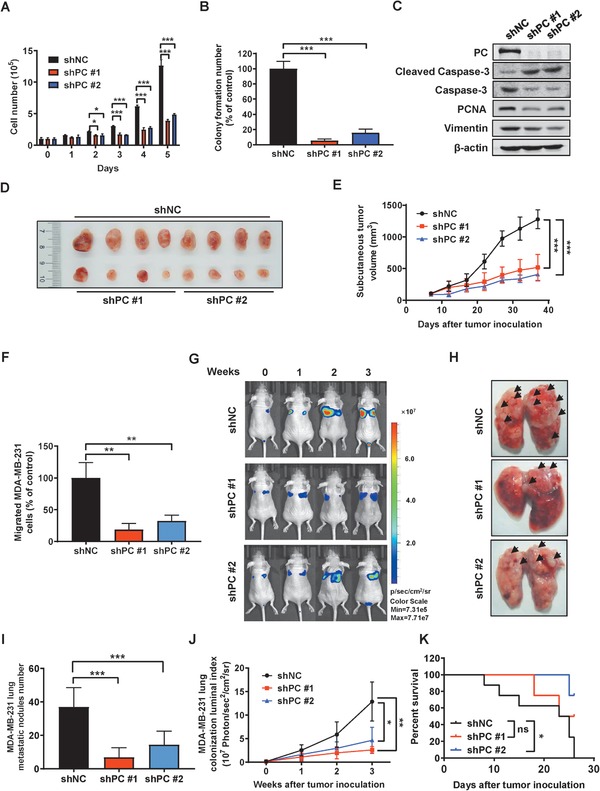
PC inhibition impaired BCA growth and lung metastases in vitro and in vivo. A) The cell numbers of shNC and shPC MDA‐MB‐231 cells were counted over 5 days. B) Colony numbers of shNC and shPC MDA‐MB‐231 cells were counted after 10 days. C) Expression of biomarkers relevant to apoptosis and migration in shNC and shPC MDA‐MB‐231 cells. D) Scrambled control cells (shNC, *n* = 8) and PC depleted MDA‐MB‐231 cells (shPC, *n* = 4 for each shRNA construct) were subcutaneously implanted in female nude mice. Resulting tumors were harvested after 37 days and are shown. E) Tumor volume progression in the shNC and shPC groups. F) The quantification of invasive cell counts of shNC and shPC MDA‐MB‐231 cells in a transwell assay. G–K) A time course of IVIS imaging was performed to detect lung colonization in the shNC group (*n* = 8) and two shPC groups (*n* = 4). G) Representative bioluminescent images were shown over three weeks. H) The arrowheads point to the visible tumor colonies in the harvested lungs. I) The numbers of lung metastatic nodules were counted. J) The whole body IVIS images provided the quantification of bioluminescent signaling from the luciferase tagged tumor cells in each group. K) The Kaplan–Meier survival curve. Data shown are mean ± s.d. *p* < 0.05, *; *p* < 0.01, **; *p* < 0.001, ***; ns, not significant.

PC catalyzes the conversion of pyruvate to oxaloacetate to replenish tricarboxylic acid cycle intermediates when depleted by excessive demand for biosynthetic purposes. Currently, little is known about PC functions in tumor growth and metastasis and PC‐associated signaling pathways. To address this problem, we performed a series of gain‐ and loss‐of‐function experiments. Two specific shRNA sequences against PC were found to efficiently downregulate PC expression in MDA‐MB‐231 cells. The scramble shRNA (shNC) cells grew in an exponential fashion whereas the two PC knockdown groups (shPC) displayed a retarded and relatively static growth over 5 days (**Figure** **5**A). PC silencing in MDA‐MB‐231 cells resulted in a more than 90% reduction of clone formation by the 10th day (Figure 5B; Figure S7A, Supporting Information). Western blotting analyses showed that PC knockdown led to higher cleaved Caspase‐3 and lower PCNA expression, biomarkers for apoptosis and proliferation, respectively (Figure 5C; Figure S7B, Supporting Information). Consistent with this, Annexin V/PI staining illustrated a marked increase in early apoptotic cells following PC knockdown (Figure S7C, Supporting Information). To evaluate the functions of PC in tumor growth in vivo, we subcutaneously implanted the same number of shNC cells and shPC cells into nude mice. After 37 days, the PC silenced cells generated much smaller tumors than control cells (Figure 5D,E), suggesting that the downregulation of PC slowed tumor growth in vivo. Thus, PC is playing a paramount role in promoting tumor growth.

### 2.13 PC Silencing Lowered BCA Metastasis In Vitro and In Vivo

We hypothesized that PC could be required for lung metastasis of BCA. We performed shRNA‐mediated PC knockdown on MDA‐MB‐231 cells and evaluated metastasis‐relevant parameters. PC downregulation resulted in lower cell migration (Figure 5F). Subsequently, an experimental intravenous mouse model was used to investigate the effects of PC knockdown on lung colonization. Quantitative luciferase signals revealed that shPC cells formed much fewer lung metastases (Figure 5G). Apparently, depleting PC expression abrogated the numbers of metastatic nodules in the lungs (Figure 5H–J). Furthermore, the median survival in a shPC group was longer than that of the shNC group (Figure 5K). Therefore, PC is essential for the expansion of lung metastases in BCA, and suppression of PC could increase the survival of mice with metastasis.

### 2.14 PC Overexpression Promoted the Proliferation and Invasion of BCA cells

To validate the essential role of PC on cancer proliferation and invasion, PC was overexpressed in the less proliferative and invasive cell line MCF7 (Figure S7D, Supporting Information). Constitutive expression of PC expedited the proliferative rate of MCF7 cells compared to empty vector controls over 5 days (Figure S7E, Supporting Information). Furthermore, the introduction of PC in MCF7 cells boosted invasiveness strongly (Figure S7F,G, Supporting Information). Collectively, PC overexpression could promote the proliferation and invasion in vitro.

### 2.15 PC Downregulation Compromised the Efficacy of ZY‐444 In Vitro

Since ZY‐444 not only bound to but also inactivated PC, we tested whether PC knockdown could influence the effects of ZY‐444 on proliferation, migration and cell metabolism.

**Figure 6:**
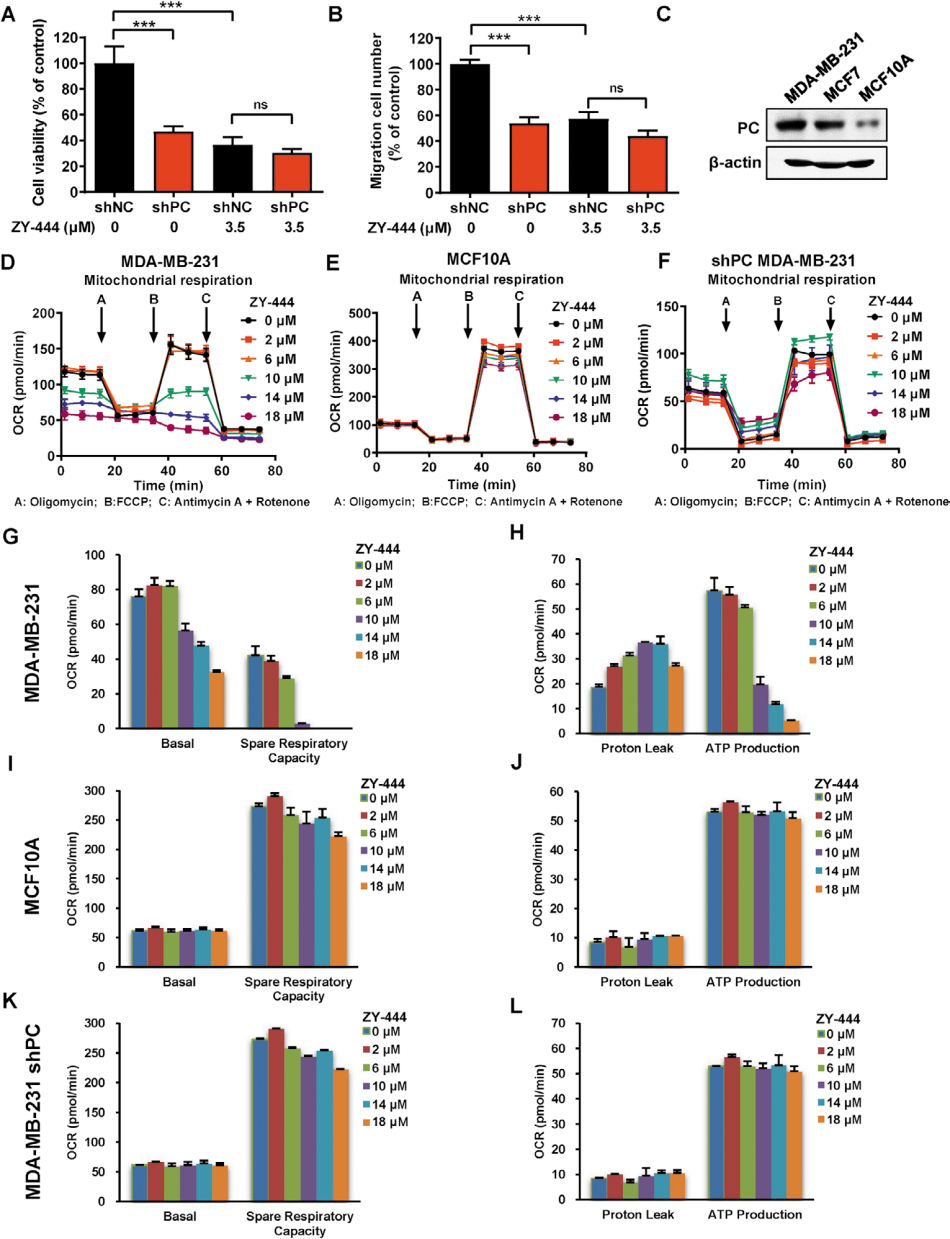
PC was required for the efficacy of ZY‐444. A) The cell viability of shNC and shPC expressing MDA‐MB‐231 cells with indicated concentrations of ZY‐444 treatment for 48 h. B) Under ZY‐444 treatment, the number of migrated shNC and shPC MDA‐MB‐231 cells was quantified. C) The relative expression of PC in BCA cells and normal breast epithelial cells. D–F) The oxygen consumption rates (OCR) were measured in cancer cells (D, MDA‐MB‐231), normal cells (E, MCF10A), and PC silenced cells (F, shPC) after ZY‐444 treatment for 5 h. G–L) Key parameters of mitochondrial respiration include basal respiration, spare respiration capacity, proton leak, and ATP production. Compared with parameters of mitochondrial respiration in G,H) MDA‐MB‐231 cells treated with ZY‐444, parameters did not change significantly in I,J) MCF10A and K,L) PC deficient MDA‐MB‐231 cells under ZY‐444 treatment. Data shown are mean ± s.d. *p* < 0.001, ***; ns, not significant.

To validate the idea that PC is indispensable for ZY‐444 efficacy, we performed both cell viability assay and wound healing assay using MDA‐MB‐231 cells expressing shRNAs targeting PC (shPC) or non‐targeted control shRNA (shNC) with or without ZY‐444 treatment. Notably, shPC cells were less sensitive to ZY‐444, indicating PC was required for a complete antiproliferation potency of ZY‐444 (**Figure** **6**A). In parral, both ZY‐444 treatment and PC knockdown obviously decreased cell migration (Figure 6B). However, on additive effect was not observed in shPC cells with ZY‐444 treatment, suggesting loss of PC expression compromised the effects of ZY‐444 on cell migration (Figure 6B). Additionally, BCA cells with higher PC expression (MDA‐MB‐231) were more sensitive to ZY‐444 than normal MCF10A cells with lower PC expression (Figures 6C and 1A,B). This may explain the selectivity of ZY‐444 for cancer cells over normal cells, which was proportional to PC expression levels.

### 2.16 ZY‐444 Prohibited Metabolism in Cancer Cells Instead of Normal Cells

Considering the differences in cytotoxicity of ZY‐444 between cancer cells versus normal cells, we hypothesized that it could be caused by cancer‐specific alterations in cell metabolism. The OCR was measured in both normal epithelial cells (MCF10A) and BCA cells (MDA‐MB‐231) exposed to ZY‐444. Exposure to ZY‐444 dose‐dependently reduced basal respiration, spare respiratory capability and ATP production in MDA‐MB‐231 cells (Figure 6D,G,H). However, the metabolic parameters of MCF10A cells did not change greatly with the same concentrations of ZY‐444 (Figures 6E,I,J). Therefore, the distinctive characteristics of cancer metabolism could contribute to the cytotoxic selectivity of ZY‐444 between cancer cells versus normal cells.

### 2.17 PC Expression Determined the Efficacy of ZY‐444 on Cell Metabolism

The mitochondrial respiration testing demonstrated that PC silencing caused metabolic alterations. Under various concentrations of ZY‐444, however, metabolic parameters remained almost unchanged in shPC cells (Figure 6F,K,L), which was similar to that in low PC expressing MCF10A cells (Figure 6I,J). These results suggested that the perturbations of mitochondrial respiration caused by ZY‐444 were dependent on PC expression. Therefore, PC is required for the pharmacological effects of ZY‐444.

### 2.18 The Expression of PC in Tumors and Normal Tissues in Cancer Patients

**Figure 7:**
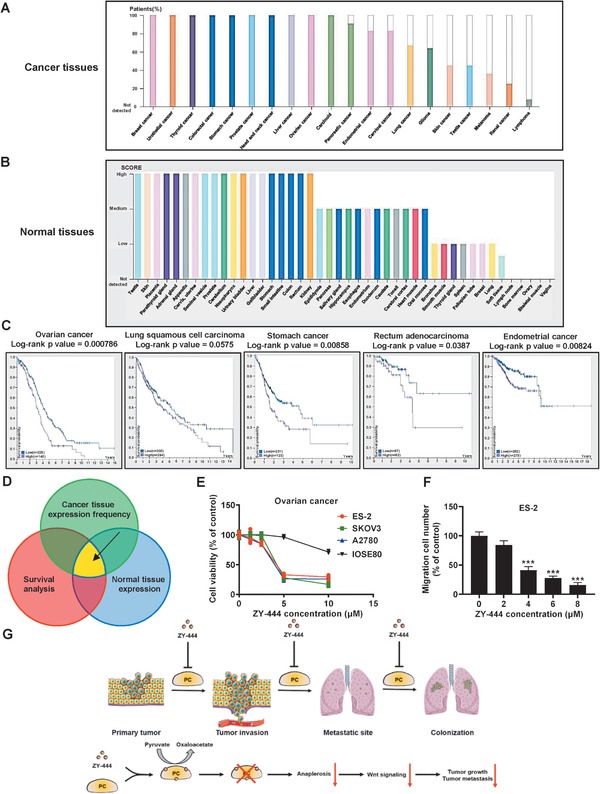
The expression of PC is associated with patient survival benefits and tumor selectivity. A) The frequency of high and medium PC expression in several types of cancer tissues from human patients. B) The PC expression scores in several types of normal human tissues. C) The Kaplan–Meier plots of patient survival segregated by high expression (purple) and low expression (blue) of PC in several types of cancer. The numbers of patients stratified by PC expression and *p*‐values using the Log‐rank test are indicated. D) Three factors were considered for potential applications of PC inhibition to achieve higher tumor selectivity, including high PC expression frequency in tumors, low normal tissue expression scores, and survival benefits in patients with lower PC expression. ZY‐444 could be potentially used in several cancer types that satisfied the three conditions above. E) Effects of ZY‐444 on proliferation in three ovarian cancer cell lines (ES‐2, SKOV3, and A2780) and an ovarian normal epithelial cell line (IOSE80). Ovarian cancer is one of the cancer types that satisfied the conditions in (D). F) Effects of ZY‐444 on migration in ovarian ES‐2 cells. G) A scheme of proposed mechanism by which ZY‐444‐mediated PC inhibition on breast cancer progression. Data shown are mean ± s.d. *p* < 0.001, ***.

Next, we investigated the expression of PC in many tumors relative to normal tissues based on The Human Protein Atlas database (THPA).^[^ ^39^ ^]^ The percentage of patients (maximum 12 patients) with high and medium PC expression levels in various cancer types is summarized in **Figure** **7**A and Table S4 (Supporting Information). PC was aberrantly and highly expressed in several tumor types (Figure S8A,B, Supporting Information). Of note, BCA solely exhibited the highest percentile (>25%) of PC amplification. To evaluate the tumor selectivity of ZY‐444 in multiple cancer types, we also examined the PC expression scores in 44 normal tissues (Figure 7B). Several normal tissues express low levels of PC. As summarized in Table S4 (Supporting Information), seven types of cancer showed higher PC frequencies in cancer tissues compared to normal tissues. This analysis implied a potential tumor selectivity of ZY‐444 in patients harboring these tumor types. Higher PC expression in breast and ovarian cancer tissues over normal tissues was confirmed by the THPA database (Figure S8C,D, Supporting Information). Subsequently, we analyzed the 5‐year survival of patients stratified by PC expression in multiple cancer types. Some cancer types presented significant differences in 5‐year survival in patients with high versus low PC expressing tumors (Figure 7C; Table S5, Supporting Information), indicating a prognostic role of PC in patient survival among several cancers.

To determine the cancer types against which PC inhibitors can possibly be effective, the relatively high tumor PC expression and PC‐related poor prognosis were considered (Figure 7D). Both the relatively higher PC expression in BCA tumors (Figure S8C, Supporting Information) and the significant correlation between higher PC expression versus lower BCA patient survival^[^ ^12^, ^40^ ^]^ indicated that ZY‐444 may show tumor selectivity in BCA patients. Moreover, among many cancer types, four cancer types (breast, ovarian, lung, and endometrial cancers) met both requirements above (Figure 7A–C),^[^ ^39^, ^41^ ^]^ implicating the possible clinical utility of ZY‐444 in these cancers.

### 2.19 ZY‐444 Treatment Was Effective in High PC Expressing Cancer Cell Lines

Among these four cancer types, we tested whether ovarian cancer cells were sensitive to ZY‐444 as ovarian cancer satisfied the requirements above. Cell proliferation and migration of three ovarian cancer cell lines (ES‐2, SKOV3, and A2780) were sensitive to ZY‐444 in vitro (Figure 7E,F). In contrast, ZY‐444 did not affect cell proliferation of human normal ovarian epithelial cells (IOSE80) (Figure 7E).

## 3 Discussion

Cumulative evidence has emerged that metabolic reprogramming is a hallmark of cancer.^[^ ^42^ ^]^ Cancer cells adapt to elevate glycolysis under aerobic contexts, a well‐appreciated phenomenon named the “Warburg effect,” to satisfy their elevated needs for the production of energy and biomass.^[^ ^1^, ^2^ ^]^ It also gives rise to the development of cancer therapeutics that function by targeting glycolysis.^[^ ^17^, ^18^, ^21^, ^42^, ^43^ ^]^ However, recent studies have expanded the notion that accelerated glycolysis is predominantly a partial accompanying mechanism for energy requirements of proliferation, and that replenishing biosynthetic macromolecules through anaplerotic pathways is vital for rapid tumor growth.^[^ ^3^, ^5^, ^44^ ^]^

PC plays a crucial role in a key anaplerotic pathway.^[^ ^8^ ^]^ Nevertheless, studies regarding PC in cancer metabolism hardly existed until 2015. Recently, researchers have discovered that PC is highly expressed in patients with NSCLC and invasive BCA.^[^ ^9^, ^10^, ^11^, ^12^ ^]^ We demonstrated higher expression of PC in multiple cancer tissues relative to corresponding non‐cancerous tissues. Importantly, high expression of PC was associated with decreased patient survival in many cancer types. All patient expression data suggest that targeting PC holds promise as a novel cancer therapy, allowing higher cancer selectivity and survival benefits.

PC participates in cancer progression in multiple ways. PC was initially identified as a mediator of anaplerosis, enabling glutamine independence of glioblastoma cells.^[^ ^45^ ^]^ Later, researchers uncovered that PC regulated cell growth and tumorigenesis by facilitating aspartate biosynthesis in cancer cells with a deficiency of succinate dehydrogenase (SDH).^[^ ^13^ ^]^ Besides NSCLC proliferation, PC‐mediated anaplerosis may be relevant to lung metastases of BCA in response to lung microenvironment.^[^ ^12^ ^]^ Our results extended the current perspectives that PC was required for BCA growth and lung metastases in vitro and in vivo (Figure 7G), and that suppression of replenishing biosynthetic macromolecule and energy supply provided by PC‐mediated anaplerosis can cause tumor regression.

Currently, the molecular mechanism of PC regulation is largely under‐exploited. The knowledge of PC regulation is largely confined to classic metabolic flux and transcriptional factors. Here, we discovered the molecular mechanism by which PC regulated canonical Wnt/β‐catenin/Snail signaling. To date, the role of the Wnt signaling pathway in cancer metabolism remains under‐investigated.^[^ ^15^ ^]^ Wnt induces anaplerosis along with increased glucose consumption and lactate production.^[^ ^14^, ^36^ ^]^ Here, we reported that PC‐deficient BCA cells also had lower canonical Wnt signaling, implicating the crosstalk between a key enzyme of anaplerosis and Wnt signaling. Given the biochemical interactions between mitochondrial enzymes and nuclear activation of Wnt signaling,^[^ ^16^ ^]^ it is possible that mitochondrial ATP deprivation caused by PC inhibition may release secondary message (Ca^2+^) to trigger ER stress followed by Wnt signaling suppression. Furthermore, it is likely that PC can also sustain phenotypes associated with Wnt signaling, such as cancer metastasis, stemness, immunity, angiogenesis and drug resistance.^[^ ^46^ ^]^ For example, the positive correlations of expression of PC and EMT markers (Snail, Slug, Vimentin, and Zeb1) existed in patient tumor samples across multiple cancer types (Figure S6D–G, Supporting Information).

Currently, most agents directly targeting cancer metabolism have been disqualified due to ineffectiveness or overt toxicity.^[^ ^47^ ^]^ To develop potent but less toxic metabolic inhibitors, we designed, synthesized and optimized a series of aryl‐heteroaromatic and pyrimidinediamine scaffold derivatives. Interestingly, most derivatives containing the “aryl‐heteroaromatic group” structure decreased mitochondrial respiration; however, the inhibitory activity disappeared when this structure was removed, suggesting its contribution to metabolic inhibition. Remarkably, we discovered a small molecule ZY‐444 that strongly and selectively inhibited cancer proliferation relative to normal cells. It reduced mitochondrial respiration and ATP production significantly and specifically in cancer cells, likely due to higher expression of a metabolic target in cancer cells. ZY‐444 bound to PC and inhibited its catalytic activity without influencing its expression. Importantly, ZY‐444 had a marked potency in cancer growth and metastasis in several preclinical models. For example, the PC inhibitor ZY‐444 remarkably suppressed the lung metastases of 4T1 tumors, in accordance with the notion that PC is indispensable for pulmonary tropism of metastatic BCA.^[^ ^12^ ^]^ Moreover, we did not observe the visible toxicity of ZY‐444 at given doses. To sum up, we identified a potent and selective metabolic inhibitor targeting PC (Figure 7G).

In addition to neoplasia, PC is related to various diseases.^[^ ^48^ ^]^ On the one hand, the fundamental roles of PC in several metabolic syndromes may suggest wider applications of ZY‐444 in multiple diseases. First, PC allows the catalyzation of the first rate‐limiting step for gluconeogenesis and equilibration of glucose produced by liver. Delivering a specific antisense oligonucleotide to decrease PC expression exclusively in liver and adipose tissue reduces gluconeogenesis, adiposity, and hepatic steatosis in rat models.^[^ ^19^ ^]^ In turn, a similar mechanism improved hepatic insulin responsiveness and reversed insulin resistance in type 2 diabetes models.^[^ ^19^, ^49^ ^]^ Second, pyruvate carboxylation by PC is the exclusive metabolic hub essential for maintaining the virulence phenotypes of *Listeria monocytogenes*, a human intracellular bacterium causing systemic infections and high fatality.^[^ ^50^ ^]^ Third, PC also potentiates the innate virus‐induced immune surveillance, suggesting another application of ZY‐444 in infection‐induced autoimmune disorders.^[^ ^51^ ^]^ Finally, PC upregulation while subjecting cells to chronic inhibition of glutamine metabolism was ascribed to the resulting resistance to therapies targeting glutaminolysis.^[^ ^45^ ^]^ ZY‐444‐based targeted therapy may circumvent the resistance to glutamine therapies found in glutamine independent cancer cells, showing promise in combination with conventional glutamine targeting treatment. On the other hand, given its suppressive PC activity, ZY‐444 at high doses can serve as a molecular tool to understand anaplerosis and PC deficiency. PC deficiency is a rare inherited disorder present at birth, creating excess blood accumulation of lactic acid.^[^ ^52^ ^]^ In most severe types of PC deficiency, patients exhibit progressive damage to their tissue and organs, particularly to the nervous system.^[^ ^53^ ^]^ The standard‐of‐care for PC deficiency is limited to providing alternative dietary sources of energy, but with limited efficacy.^[^ ^53b^ ^]^ High doses of ZY‐444 may be utilized in preclinical models to mimic the conditions of PC deprivation, which may help understand the mechanism of PC deficiency and develop appropriate and effective therapies that are not available at present.

## 4 Conclusion

Our study provides a mechanistic perspective of a key anaplerotic enzyme PC, and identified a potent and selective small molecule, ZY‐444, as an inhibitor of PC. We also examined the possibilities of clinical investigation in multiple cancer types and PC‐associated diseases. As a lead compound with aryl‐heteroaromatic groups, ZY‐444 can be further investigated for developing novel agents of metabolic regulation.

## 5 Experimental Section

The Experimental Section is available in the Supporting Information.

## Conflict of Interest

The authors declare no conflict of interest.

## Author Contributions

Q.L. and Y.H. contributed equally to this work. Q.L., Y.H., X.W., Z.S., Z.Y., M.L., and Y.C. designed the experiments. Q.L., Y.H., X.W., Y.Z., M.H., W.G., Y.H., T.Z., and L.L. performed the experiments. Q.L., Y.H., X.W., M.H., W.G., Z.S., Z.Y., M.L., and Y.C. performed the data analysis. Q.L., Y.H., Z.Y., M.L., and Y.C. wrote the manuscript.

## Supporting information

Supporting Information
