# Supplementary material for: Targeting Pyruvate Carboxylase by a Small Molecule Suppresses Breast Cancer Progression
Source: Adv Sci (Weinh). 2020 Mar 12;7(9):1903483. doi: 10.1002/advs.201903483 (PMC7201266; doi:10.1002/advs.201903483)
Supplement: Supplementary file 1 — Supporting Information [file ADVS-7-1903483-s001.pdf]

Copyright WILEY-VCH Verlag GmbH & Co. KGaA, 69469 Weinheim, Germany, 2020.

Supporting Information

for *Adv. Sci.,* DOI: 10.1002/advs.201903483

Targeting Pyruvate Carboxylase by a Small Molecule Suppresses Breast Cancer Progression

Qingxiang Lin, Yuan He, Xue Wang, Yong Zhang, Meichun Hu, Weikai Guo, Yundong He, Tao Zhang, Li Lai, Zhenliang Sun, Zhengfang Yi, Mingyao Liu,* and Yihua Chen**

Supporting Information

Targeting Pyruvate Carboxylase by a Small Molecule Suppresses Breast Cancer Progression

Qingxiang Lin, Yuan He, Xue Wang, Yong Zhang, Meichun Hu, Weikai Guo, Yundong He, Tao Zhang, Li Lai, Zhenliang Sun, Zhengfang Yi, Mingyao Liu,* and Yihua Chen**

1. Materials and Methods

Synthesis of N^4-((5-(4-(benzyloxy)phenyl)-2-thiophenyl)methyl)-N^2-isobutyl-2,4-pyrimidine-diamine (ZY-444):

To a solution of compound *N*-((5-(4-(benzyloxy)phenyl)-2-thiophenyl)methyl)-2-chloro-4-pyrimidinamine (729 mg, 1.79 mmol) and isobutylamine (1.80 ml, 17.9 mmol) in *n*-BuOH (10 ml), and then DIEA (0.92 ml, 5.37mmol) was added, and the resulting mixture was stirred at 120 °C for 8 h. Then the reaction mixture was concentrated under reduced pressure and extracted with CH_2Cl_2 and H_2O. The organic layer was washed with brine and dried over anhydrous Na_2SO_4, and concentrated. The residue was purified by column chromatography (PE/EtOAc 2:1 followed by 80:1 to 20:1 CH_2Cl_2/MeOH) to give N^4-((5-(4-(benzyloxy)phenyl)-2-thiophenyl)methyl)-N^2-isobutyl-2,4-pyrimidine-diamine (ZY-444). ^{1}H NMR (500 MHz, DMSO-d_6) δ 7.66 (d, J = 5.9 Hz, 1H), 7.50 (d, J = 8.8 Hz, 2H), 7.45 (d, J = 7.2 Hz, 2H), 7.40 (t, J = 7.4 Hz, 2H), 7.33 (t, J = 7.2 Hz, 1H), 7.20 (d, J = 3.6 Hz, 1H), 7.03 (d, J = 8.8 Hz, 2H), 6.97 (d, J = 3.6 Hz, 1H), 5.81 (d, J = 6.0 Hz, 1H), 5.13 (s, 2H), 4.63 (d, J = 3.4 Hz, 2H), 3.19 – 2.99 (m, 2H), 1.88 – 1.79 (m, 1H), 0.87 (d, J = 6.7 Hz, 6H). ^{13}C NMR (125 MHz, DMSO-d_6) δ 158.29, 142.66, 137.40, 128.89, 128.31, 128.14, 127.30, 127.12, 126.86, 122.25, 115.82, 69.74, 48.64, 28.32, 20.69. HR MS (ESI): calcd for $C_{26}H_{29}N_4O_S$ $[M + H]^+$: 445.2057; found 445.2053 (Figure S4A, Supporting Information).

Cell Lines and Cell Culture

Information on cancer cell lines is shown in Table S6. Mouse breast tumor 4T1 cells were transfected with pGL4 vector (Promega Madison, WI, USA) and selected in G418 to generate a stable 4T1-luc cell line. All cell lines were maintained in recommended cell culture media and conditions.

Reagents and Antibodies

Detailed information on antibodies is shown in Table S7. Purified PC (9014-19-1) and paclitaxel (T7402) were purchased from Sigma. The PC activity kit was obtained from Comin Biotechnology Co., Ltd. (Suzhou, China).

Animals

All mice were maintained in pathogen-free conditions. Animal studies were performed according to the guidelines approved by the Institute of Biomedical Sciences, East China Normal University.

Seahorse Cell Mito Stress Test

The XF Cell Mito Stress Test (Seahorse Bioscience, MA, USA) uses modulators of respiration that target components of the electron transport chain (ETC) in the mitochondria to measure key parameters of metabolic functions.[1] The oxygen consumption rate (OCR) was measured using a Seahorse XF-96 analyzer. Before OCR measurement, the sensor cartridge was calibrated with calibration buffer at 37 °C in a non-CO_2 incubator overnight. Cells were plated at an optimized density in an XF Cell Culture Microplate. On the measurement day, cells were incubated with fresh medium containing different concentrations of compounds for 5 h. Cells were washed with XF assay medium (pH 7.4) supplemented with 1 mM pyruvate, 2 mM glutamine, and 10 mM glucose and placed in a 37 °C incubator without CO_2 for 1 h before calibration. 2 μM oligomycin, 1 μM protonophoric uncoupler (FCCP) and 0.5 μM antimycin A + rotenone were preloaded in reagent Ports-A, B and C. Three readings were taken after the addition of each reagent prior to injection with subsequent reagents. OCR was automatically calculated and recorded by the sensor cartridge and Seahorse XF-96 software. The numerical data of samples in the test were standardized against the cell number.

Cell Viability Assay

As previously described, [2] 5×10^3 cells were seeded in 96-well plates and a range of concentrations of drugs was added next day for 48 h. One Solution (Promega) was added to measure the absorption at 490 nm with a microplate spectrophotometer.

Apoptosis Assay

As previously described,[3] cells were treated with different concentrations of drugs. The cell pellets were washed with PBS and re-suspended in binding buffer containing FITC-conjugated Annexin V and PI (BD Biosciences). Flow cytometer (BD Biosciences) was utilized to determine the cell apoptosis.

Wound Healing Migration Assay

As previously reported,[4] wound healing assay was performed in 6-well plates. Confluent cells were starved overnight and scratched. Cells were cultured in indicated concentrations of drugs for 12-24 h and fixed with 4% paraformaldehyde. Migrated cells were imaged by an inverted microscope (Olympus) and counted manually.

Boyden Transwell Invasion Assay

Invasion assay was conducted using transwell chambers (Millipore) with 8-μm pore membrane pre-coated with 100 μl Matrigel (BD Biosciences).[5] In the upper chambers, 5×10^3 cells were seeded along with indicated concentrations of drugs in serum-free medium. Serum-containing medium was placed in each bottom well. Non-invasive cells on the upper chambers were removed using cotton swabs 12-16 h later. Invaded cells on the lower chambers were fixed with 5% paraformaldehyde and stained with 0.2% crystal violet. Images were taken by an inverted microscope (Olympus) and invaded cell numbers were quantified manually.

Immunofluorescent Staining

Cells were plated on glass coverslips and subjected to treatment. The cells were then fixed with 4% paraformaldehyde followed by permeabilization using Triton X-100 (0.2%) for 5 min. The cells were subsequently washed with PBS, blocked with 3% BSA for 1 h, and then incubated with primary antibody at 4 °C overnight. Later, the cells were incubated with a secondary antibody (Invitrogen) for 1 h, and then the nuclei were counterstained with DAPI for 5 min. The images were acquired using a confocal microscope (Leica).

Nuclear and Cytoplasmic Extraction

Cells were lysed using Nuclear and Cytoplasmic Protein Extraction Kit (Sangon Biotech, C51001) in accordance with the manufacturer's protocol.

Western Blot Analysis

As described previously, [3] Western blot analysis was performed. Briefly, samples were prepared with cell lysis buffer containing proteinase inhibitor cocktail (EMD Millipore) and 1 mM phenylmethylsulfonyl fluoride (PMSF, Cell Signaling Technology). The amounts of total protein were quantified by BCA assay kit (Thermo Fisher) for equal loading. The loaded samples were electrophoresed by SDS-PAGE and transferred to PVDF membranes. Membranes were scanned (Odyssey, LI-COR) after incubation with primary and relevant secondary antibodies; see Supplementary Table 7 for antibody details.

Spontaneous Breast Cancer Metastasis Mouse Models

Approximately 5×10^4 4T1-luc cells in 50 µl PBS were injected into the fourth (abdominal) mammary gland into 4-week-old female BALB/c mice.[6] On the 6th day, mice were divided into several groups (n = 6-10) with similar tumor burden for treatment. 1) DMSO vehicle; 2) 2.5 mg/kg ZY-444; 3) 5.0 mg/kg ZY-444; 4) 5.0 mg/kg paclitaxel. All agents were administered *via*

intraperitoneal injection (i.p.) daily. The tumor volume was measured and calculated using the formula: volume = length × width^2 × 0.52. After treatment over 21 days, metastases were determined using bioluminescent imaging on a Xenogen IVIS-200 (Caliper Life Sciences). Then all the mice were sacrificed, and tumors and organs (lungs, liver, heart, leg bone, spleen and kidney) were imaged to detect luciferase signals. The 4T1-luc tumor metastases in lungs were visualized and counted.

Similarly, 6-week-old female nude mice were implanted with 1×10^6 MDA-MB-231 cells expressing luciferase (Caliper Life Sciences) in the fourth mammary fat pad and then allocated into 4 groups (n = 7) with indicated doses: 1) DMSO vehicle; 2) 2.5 mg/kg ZY-444; 3) 5 mg/kg ZY-444; 4) 5 mg/kg paclitaxel. Drugs were dosed i.p. every two days after implantation. Tumor size and body weight were recorded over the 28 days of treatment. Tumors and indicated organs were imaged and quantified with IVIS.

Experimental Breast Cancer Lung Colonization Mouse Model

One million MDA-MB-231-luc cells in 100 µl PBS were inoculated into 6-week-old female nude mice intravenously.[7] The mice were segregated into four groups according to the initial average IVIS images. Drug administration was conducted every two days with the following doses. 1) DMSO vehicle (n = 12); 2) 2.5 mg/kg ZY-444 (n = 11); 3) 5 mg/kg ZY-444 (n = 12); 4) 5 mg/kg paclitaxel (n = 12). On the last day, luciferase signaling was acquired using the Xenogen IVIS and quantified. Kaplan-Meier survival curve was made over the treatment periods.

Adjuvant Therapeutic Metastasis Mouse Model

An adjuvant therapeutic metastasis mouse model was modified from a previous study [8]. 4T1-luc cells were implanted into mammary gland of female BALB/c mice. On the 14th day, tumors were palpable and lung metastasis had started. Mice were then separated into four groups. Subsequently,

the primary tumors were completely removed surgically, and the wound was sutured carefully. After two days, agents were injected i.p. daily over 2 weeks as follows: 1) DMSO vehicle (n = 10); 2) 2.5 mg/kg ZY-444 (n = 8); 3) 5 mg/kg ZY-444 (n = 8); 4) 5 mg/kg paclitaxel (n = 8). Tumor recurrence and distant metastases were detected on the 31st day using bioluminescence imaging and the recurrence incidence was calculated. In parallel, mice survival was monitored, and Kaplan-Meier survival analysis was performed.

Biotin-assisted Pull-down Assay and Mass Spectrometry Analysis

MDA-MB-231 cells were lysed using Mg^{2+} lysis/wash buffer (25 mM, HEPES, pH 7.5, 150 mM NaCl, 1% Igepal CA-630, 10 mM $MgCl_2$, 1 mM EDTA and 2% glycerol) and subjected to protein quantification. Whole cell protein extracts (500 μg) were incubated with 10 μM biotin or biotin-ZY-444 as well as excess streptavidin-agarose beads (Thermo Inc.) at 4 °C overnight. Next, the bead-bound proteins were eluted, separated by SDS-PAGE and visualized by silver staining (Sigma). The precipitated bands in the gel that contained the target protein were excised carefully, followed by in-gel digestion and analysis by LC-MS/MS.[9]

Surface Plasmon Resonance (SPR) Binding Assay

As previously described,[5] Surface Plasmon Resonance (SPR) analysis was performed with a Biacore T200 instrument (GE Healthcare). Compound biotin-ZY-444 (10 μM) in PBS buffer (pH 7.4) was immobilized on the sensor chip (SA). To remove any remaining unbound compounds, the surface of the flow cell was then washed with PBS buffer. Pure PC protein (1.25 nM - 40 nM) dissolved in degassed and filter-sterilized PBS buffer was injected into the channel and analyzed at 25 °C. The resulting binding response curves were double reference subtracted. Data processing and analysis were performed using BIAevaluation software in a 1:1 Langmuir binding model.

Pyruvate Carboxylase Activity Assay

PC activity was performed following the PC kit instructions (Comin Biotechnology Co., Ltd. Suzhou, China). Briefly, MDA-MB-231 cells were treated with DMSO or a range of concentrations of ZY-444 for 24 h. Then 5×10^6 cells were washed with PBS, 1 ml extract buffer (50 mM Tris-HCl, pH 7.4 containing 2 mM magnesium acetate, 1 mM EDTA and 10% glycerol) was added, cells were collected and lysed by homogenizer in an ice bath. Insoluble particles were removed by differential centrifugation (600 g, 5 min, 4 °C). The supernatant was transferred to another micro-tube and centrifuged at 11000 g for 10 min at 4 °C. Next, the supernatant was removed, and the precipitate was suspended in 1 ml extract buffer and sonicated at 200 W output in an ice bath with 30 × 3 sec pulses with 10 sec intervals. Then the working solution was prepared by mixing malate dehydrogenase, acetyl coenzyme and NADH and then incubated for 5 min at 37 °C. The reaction solution containing 10 μl sample, 10 μl reagent (100 mM triethanolamine buffer pH 8.0 containing 30 mM ATP and 450 mM sodium bicarbonate) and 180 μl working solution were combined in a 96-well plate. Two minutes later, the absorbance A1 and the absorbance A2 were detected by a microplate reader. The values of PC activity (nmol/min/10^4 cells) were calculated as 6.43 × (A1 - A2).

shRNA Mediated PC Knockdown Assay

shRNAs against human PC were inserted into the pLKO.1 vector. HEK 293T cells were transfected with the following plasmids: lentiviral packaging plasmids PMD2G, PSPAX2 and lentiviral expression plasmid containing shRNA against PC (shPC #1 or shPC #2). The target sequences for shRNAs were as follows:

Scrambled shRNA (shNC): 5'-TTCTCCGAACGTGTCACGTTT-3'.

shPC #1: 5'-GCCCAGTTTATGGTGCAGAAT-3';

shPC #2: 5'-GCCAAGGAGAACAACGTAGAT-3'.

After 72 h, medium from the transfected cells was collected, filtered, supplemented with 10 μg/ml polybrene, and added to MDA-MB-231 cells in an equal volume of DMEM media. The MDA-MB-231 cells bearing the plasmid with the puromycin resistance gene were selected using 1 μg/ml puromycin.

Subcutaneous Tumor Model

Same numbers of scrambled and shPC (shPC #1 or shPC #2) MDA-MB-231 cells were injected subcutaneously into female nude mice. Tumor volumes were measured over a month. The scrambled (n = 8) and shPC (shPC #1 and shPC #2, n = 4 for each) tumors were excised and imaged on the 37th day.

Overexpression of PC Assay.

The 3 μg empty control vector or pcDNA3.1-PC (NM_000920, Catalog: F118260, Youbio Biotechnology, China) vector was transfected into MCF7 cells using 6 μl Lipofectamine TM 2000 reagent (Invitrogen) in 6-well plates according to the manufacturer's instructions. After 48 h of transfection and puromycin resistance screening, the expression of PC was tested.

Microarray Assay and Analysis

MDA-MB-231 cells were plated in a 6-well plate for 24 h and then incubated in 5 μM ZY-444 for 24 h. Next, cells were harvested using Trizol Reagent (Invitrogen Carlsbad), and mRNA microarray analysis was conducted by Affymetrix GeneChip U133 plus 2.0 Array (Affymetrix, Santa Clara, CA) according to the manufacturer's instructions. Fluorescent signaling was scanned with an Affymetrix GeneChip Scanner 3000. Data collected were analyzed in R software and Panther database.[10]

Real-time Quantitative PCR

Total RNA for various concentrations of ZY-444 was extracted with Trizol reagent (Invitrogen) and reverse transcribed into cDNA using PrimeScript RT reagent Kit (TaKaRa Bio; Tokyo, Japan). RT-PCR was conducted using the following procedures: 1 cycle for 5 s at 95 °C; 35 cycles for 30 s at 94 °C, 55 °C and 72 °C, respectively; 1 cycle for 30 s at 94 °C, 55 °C and 94 °C respectively. The sequences of primers are shown as follows.

PC Forward：GCGTGTTTGACTACAGTGAG;

PC Reverse：TCTTGACCTCCTTGAACTTG.

β-actin Forward：CATGTACGTTGCTATCCAGGC;

β-actin Reverse：CTCCTTAATGTCACGCACGAT

Data Sources of Patient Samples

The gene alteration frequency and RNAseq data of PC expression in different tumor types were derived from cBioPortal for Cancer Genomics. The expression of PC in several cancers *vs.* normal tissues was retrieved from The Human Protein Atlas.[11] The Kaplan Meier plots were obtained from The Human Protein Atlas. The results of protein co-expression in patient tumors were analyzed using Gene Expression Profiling Interactive Analysis (GEPIA).[12]

Statistical Analysis

For most cell experiments, three independent experiments were conducted in triplicate. Results are shown as mean ± SD. P-values less than 0.05 were considered statistically significant. Group-wise comparisons were determined by two-sided, unpaired Student's t-test. One-way ANOVA or Two-way ANOVA was performed for multiple comparisons. Survival data were compared by Kaplan Meier survival analysis using the Log-rank (Mantel-Cox) test.

References

[1] E. Carbognin, R. M. Betto, M. E. Soriano, A. G. Smith, G. Martello, *EMBO J.* **2016**, *35*, 618.

[2] W. Zhou, A. Huang, Y. Zhang, Q. Lin, W. Guo, Z. You, Z. Yi, M. Liu, Y. Chen, *Eur. J. Med. Chem.* **2015**, *96*, 269.

[3] X. Wang, Q. Lin, F. Lv, N. Liu, Y. Xu, M. Liu, Y. Chen, Z. Yi, *Leukemia* **2016**, *30*, 1465.

[4] L. Lai, J. Liu, D. Zhai, Q. Lin, L. He, Y. Dong, J. Zhang, B. Lu, Y. Chen, Z. Yi, M. Liu, *Brit. J. Pharmacol.* **2012**, *165*, 1084.

[5] T. Zhang, J. Li, Y. He, F. Yang, Y. Hao, W. Jin, J. Wu, Z. Sun, Y. Li, Y. Chen, Z. Yi, M. Liu, *Nat. Commun.* **2018**, *9*, 3726.

[6] C. Zheng, Y. Fang, W. Tong, G. Li, H. Wu, W. Zhou, Q. Lin, F. Yang, Z. Yang, P. Wang, Y. Peng, X. Pang, Z. Yi, J. Luo, M. Liu, Y. Chen, *J. Med. Chem.* **2014**, *57*, 600.

[7] T. Zhang, J. Li, X. Ma, Y. Yang, W. Sun, W. Jin, L. Wang, Y. He, F. Yang, Z. Yi, Y. Hua, M. Liu, Y. Chen, Z. Cai, *EBioMedicine* **2018**, *31*, 276.

[8] T. Zhang, Y. Chen, J. Li, F. Yang, H. Wu, F. Dai, M. Hu, X. Lu, Y. Peng, M. Liu, Y. Zhao, Z. Yi, *Neoplasia* **2014**, *16*, 665.

[9] C. X. Liu, Q. Q. Yin, H. C. Zhou, Y. L. Wu, J. X. Pu, L. Xia, W. Liu, X. Huang, T. Jiang, M. X. Wu, L. C. He, Y. X. Zhao, X. L. Wang, W. L. Xiao, H. Z. Chen, Q. Zhao, A. W. Zhou, L. S. Wang, H. D. Sun, G. Q. Chen, *Nat. Chem. Biol.* **2012**, *8*, 486.

[10] a) P. D. Thomas, M. J. Campbell, A. Kejariwal, H. Mi, B. Karlak, R. Daverman, K. Diemer, A. Muruganujan, A. Narechania, *Genome Res.* **2003**, *13*, 2129; b) P. D. Thomas, A. Kejariwal, N. Guo, H. Mi, M. J. Campbell, A. Muruganujan, B. Lazareva-Ulitsky, *Nucleic Acids Res.* **2006**, *34*, W645.

[11] a) M. Uhlen, E. Bjorling, C. Agaton, C. A. Szigyarto, B. Amini, E. Andersen, A. C. Andersson, P. Angelidou, A. Asplund, C. Asplund, L. Berglund, K. Bergstrom, H. Brumer, D. Cerjan, M. Ekstrom, A. Elobeid, C. Eriksson, L. Fagerberg, R. Falk, J. Fall, M. Forsberg, M. G. Bjorklund, K. Gumbel, A. Halimi, I. Hallin, C. Hamsten, M. Hansson, M. Hedhammar, G. Hercules, C. Kampf, K. Larsson, M. Lindskog, W. Lodewyckx, J. Lund, J. Lundeberg, K. Magnusson, E. Malm, P. Nilsson, J. Odling, P. Oksvold, I. Olsson, E. Oster, J. Ottosson, L. Paavilainen, A. Persson, R. Rimini, J. Rockberg, M. Runeson, A. Sivertsson, A. Skollermo, J. Steen, M. Stenvall, F. Sterky, S. Stromberg, M. Sundberg, H. Tegel, S. Tourle, E. Wahlund, A. Walden, J. Wan, H. Wernerus, J. Westberg, K. Wester, U. Wrethagen, L. L. Xu, S. Hober, F. Ponten, *Mol. Cell Proteomics* **2005**, *4*, 1920; b) F. Ponten, K. Jirstrom, M. Uhlen, *J. Pathol.* **2008**, *216*, 387; c) L. Berglund, E. Bjorling, P. Oksvold, L. Fagerberg, A. Asplund, C. A. Szigyarto, A. Persson, J. Ottosson, H. Wernerus, P. Nilsson, E. Lundberg, A. Sivertsson, S. Navani, K. Wester, C. Kampf, S. Hober, F. Ponten, M. Uhlen, *Mol. Cell Proteomics* **2008**, *7*, 2019; d) M. Uhlen, C. Zhang, S. Lee, E. Sjostedt, L. Fagerberg, G. Bidkhori, R. Benfeitas, M. Arif, Z. Liu, F. Edfors, K. Sanli, K. von Feilitzen, P. Oksvold, E. Lundberg, S. Hober, P. Nilsson, J. Mattsson, J. M. Schwenk, H. Brunnstrom, B. Glimelius, T. Sjoblom, P. H. Edqvist, D. Djureinovic, P. Micke, C. Lindskog, A. Mardinoglu, F. Ponten, *Science* **2017**, *357*; e) P. J. Thul, L. Akesson, M. Wiking, D. Mahdessian, A. Geladaki, H. Ait Blal, T. Alm, A. Asplund, L. Bjork, L. M. Breckels, A. Backstrom, F. Danielsson, L. Fagerberg, J. Fall, L. Gatto, C. Gnann, S. Hober, M. Hjelmare, F. Johansson, S. Lee, C. Lindskog, J. Mulder, C. M. Mulvey, P. Nilsson, P. Oksvold, J. Rockberg, R. Schutten, J. M. Schwenk, A. Sivertsson, E. Sjostedt, M. Skogs, C. Stadler,

D. P. Sullivan, H. Tegel, C. Winsnes, C. Zhang, M. Zwahlen, A. Mardinoglu, F. Ponten, K. von Feilitzen, K. S. Lilley, M. Uhlen, E. Lundberg, *Science* **2017**, *356*.

[12] Z. Tang, C. Li, B. Kang, G. Gao, C. Li, Z. Zhang, *Nucleic Acids Res.* **2017**, *45*, W98.

2. Supplementary Figures

Lin *et al.* Figure S1

A

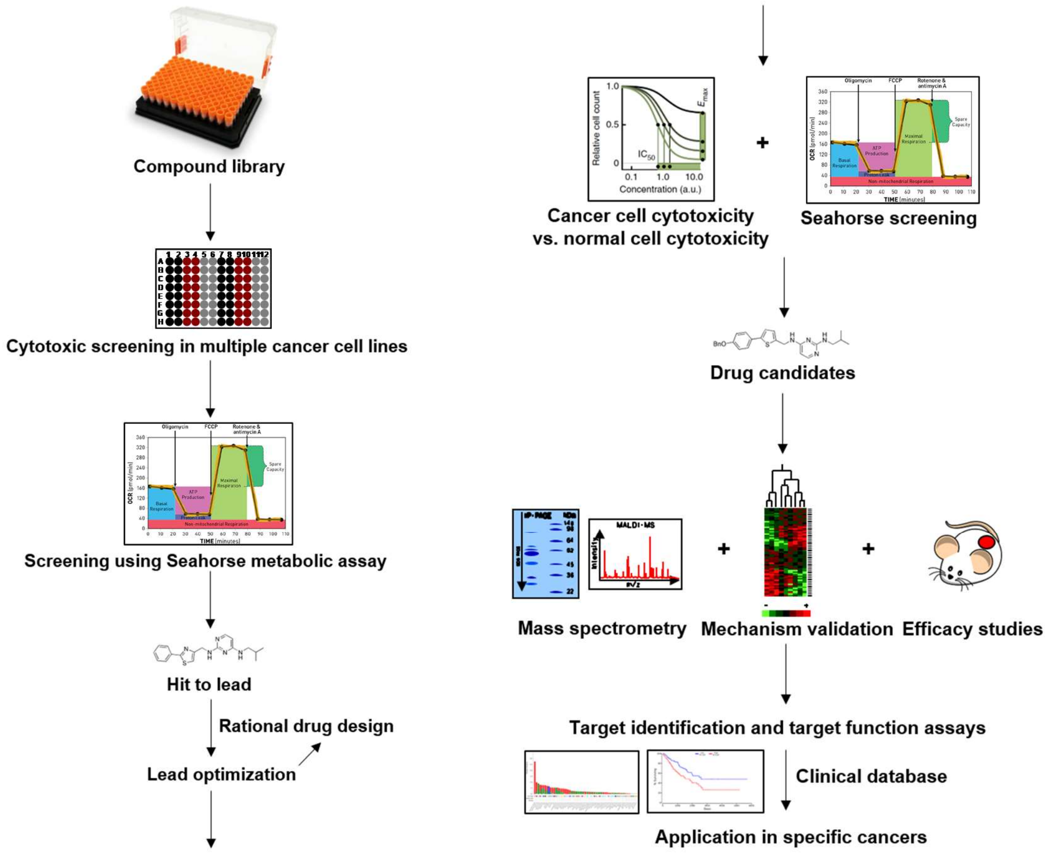

B

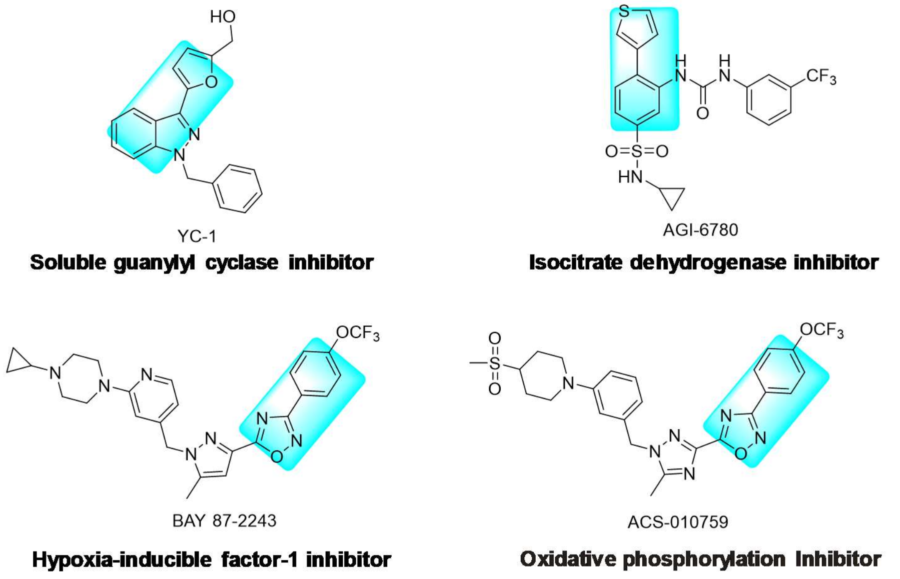

Lin *et al.* Figure S1

C

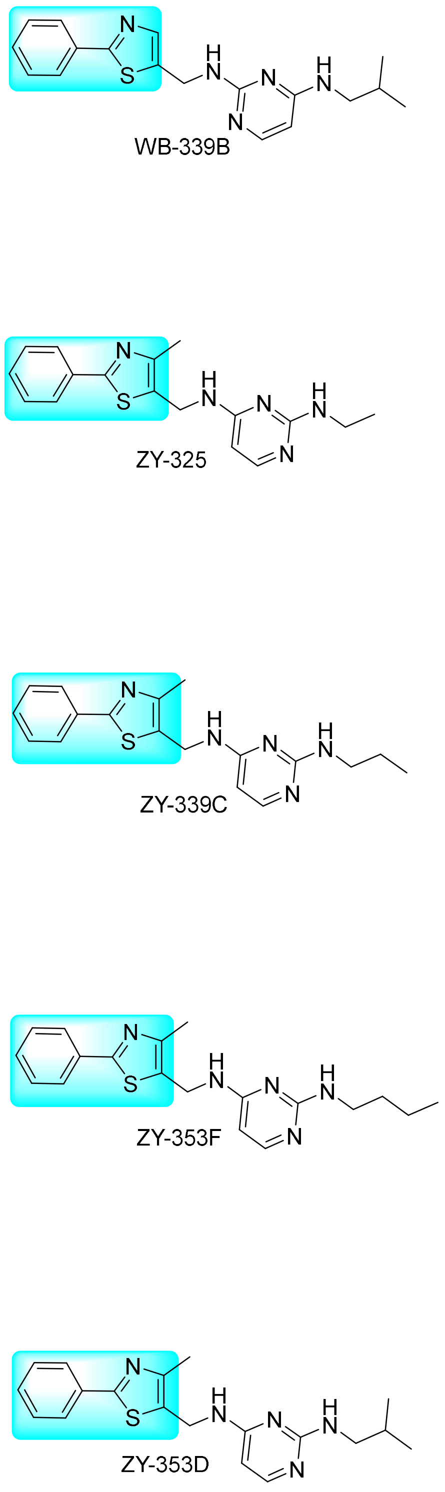

D

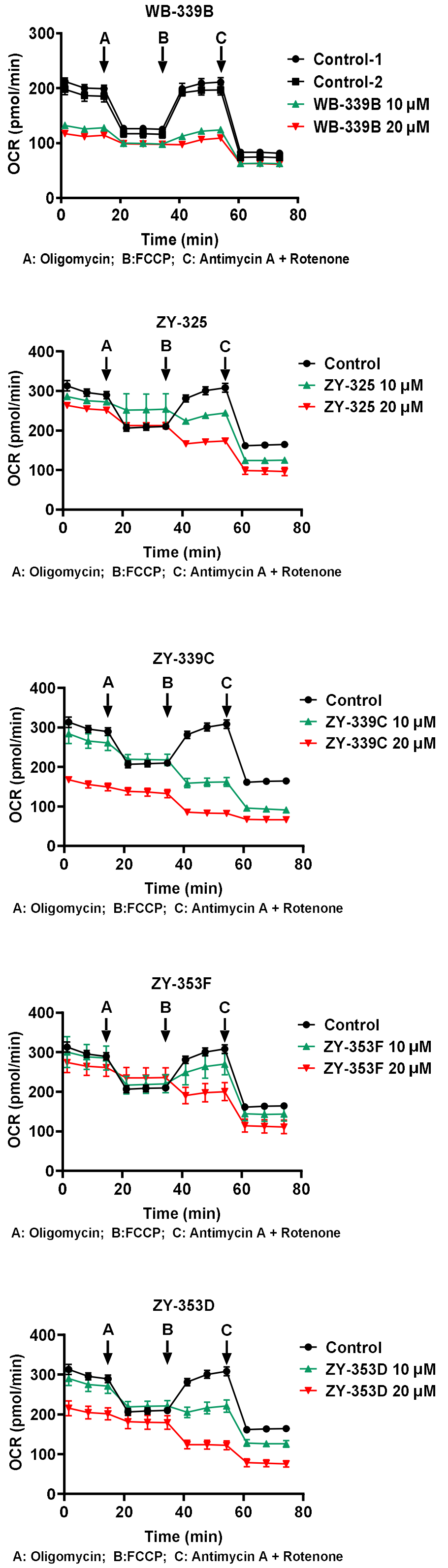

Lin *et al.* Figure S1

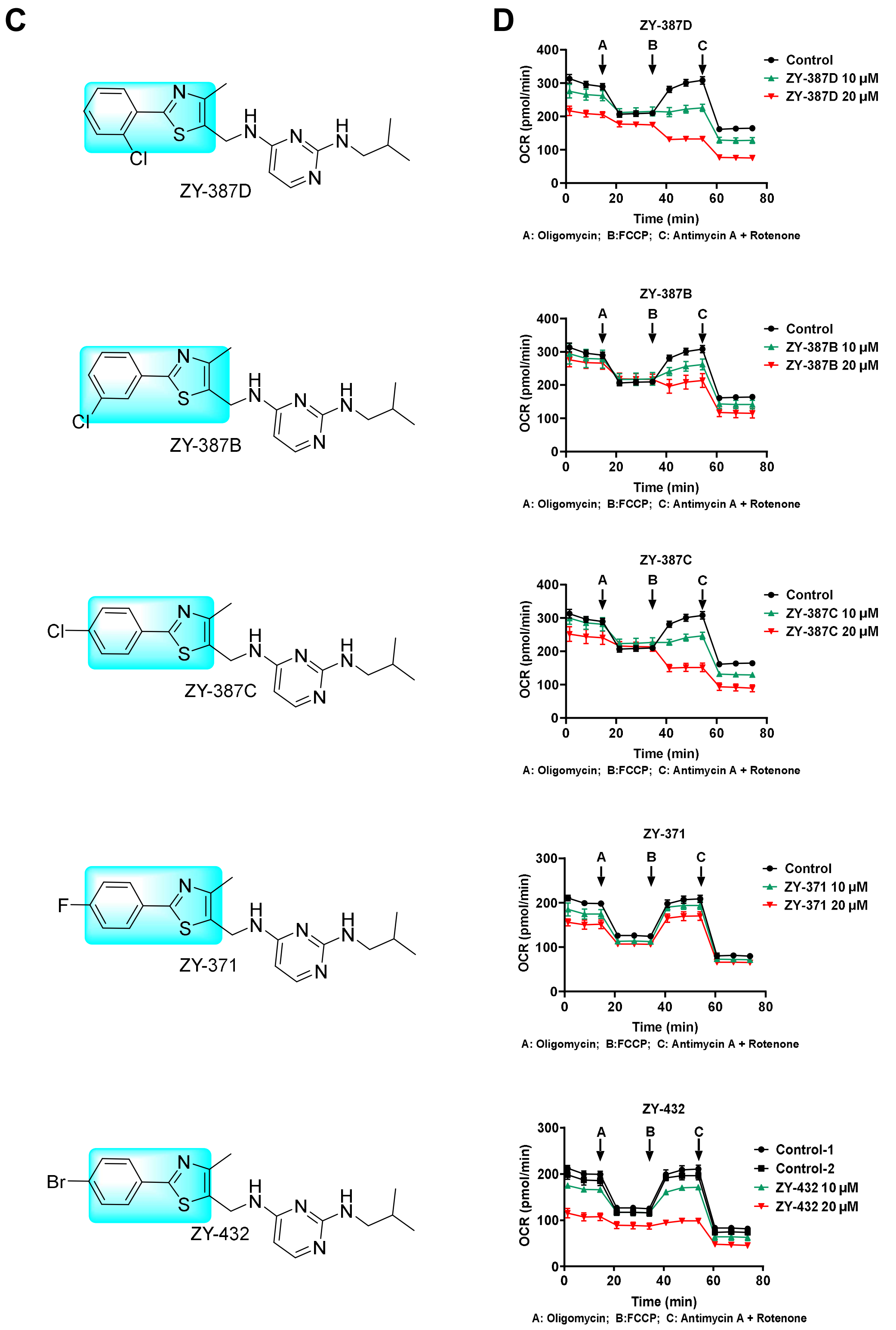

Lin *et al.* Figure S1

C

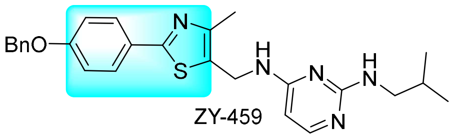

ZY-459

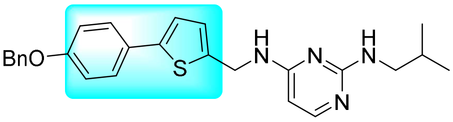

ZY-444

D

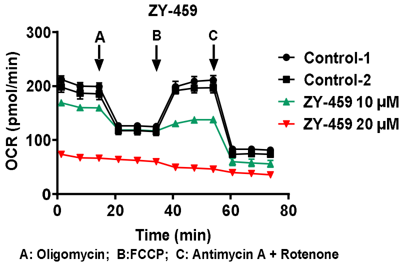

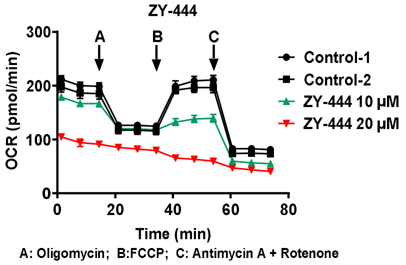

E

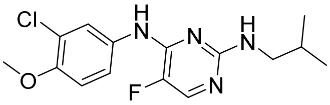

WK-325

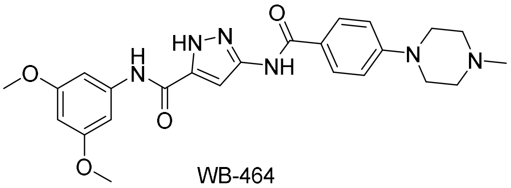

WB-464

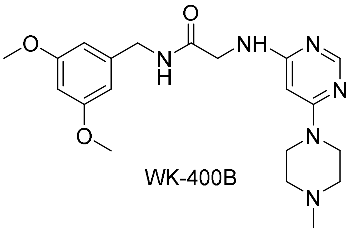

WK-400B

F

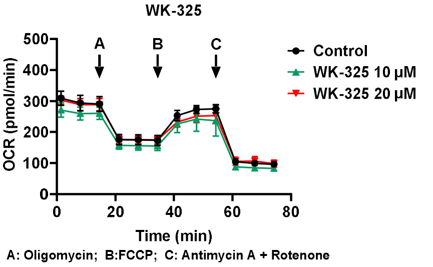

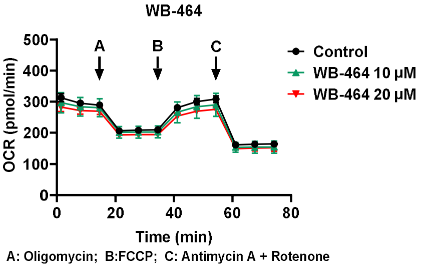

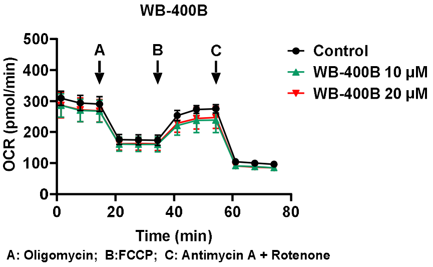

Figure S1. Compounds sharing similar structures show inhibition of MDA-MB-231 mitochondrial respiration capabilities.

A) Workflow of discovery of cancer metabolism modulators. Lead compounds that potentially inhibited mitochondrial respiration were screened by cell proliferation assay followed by Seahorse XF Cell Mito Stress test. Hits to lead were then optimized chemically. Derivatives were subjected to cytotoxic assays in normal cells and cancer cells, as well as the Seahorse XF Cell Mito Stress test. The candidate modulators of cancer metabolism were identified based on the extent of selectivity for cancer cells over normal cells and inhibition of mitochondrial respiration. ZY-444 was selected for further pharmacological investigation.

B) Chemical structures of known metabolic inhibitors. The highlighted structure (aryl-heteroaromatic groups) was hypothesized to contribute to their inhibition of metabolic capability.

C-F) Chemical structures of many WB-339B derivatives (C, left) and respective inhibition of mitochondrial respiration (D, right) using the Seahorse Mito Stress test. The chemical structures of negative compounds lacking aryl-heteroaromatic groups and corresponding activities on inhibition of mitochondrial respiration are shown in (E) and (F).

Lin *et al.* Figure S2

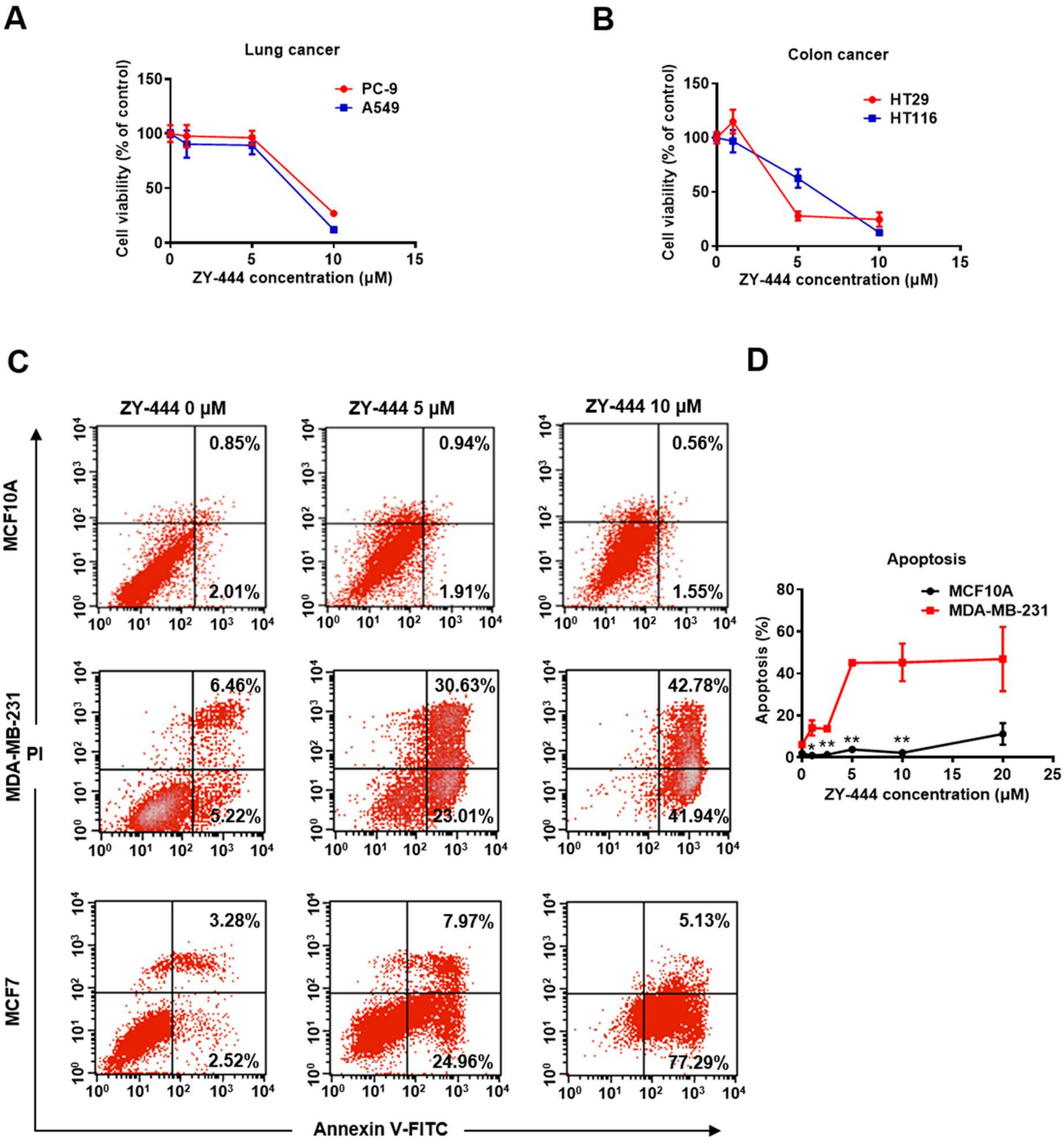

Figure S2. ZY-444 induces apoptosis in cancer cells but not in normal epithelial cells.

A-B) Cell viability of several lung (A), and colon cancer (B) cell lines under ZY-444 treatment for 48 h.

C) Apoptotic induction by ZY-444 treatment of MCF10A, MDA-MB-231 and MCF7 cells for 12 h. The percentiles of apoptotic induction at early and late stages are shown in the top right and bottom right quadrants.

D) Quantification of apoptotic induction in MDA-MB-231 and MCF10A cells using a range of concentrations of ZY-444.

Data shown are mean ± s.d. $p < 0.05$, *; $p < 0.01$, **.

Lin *et al.* Figure S3

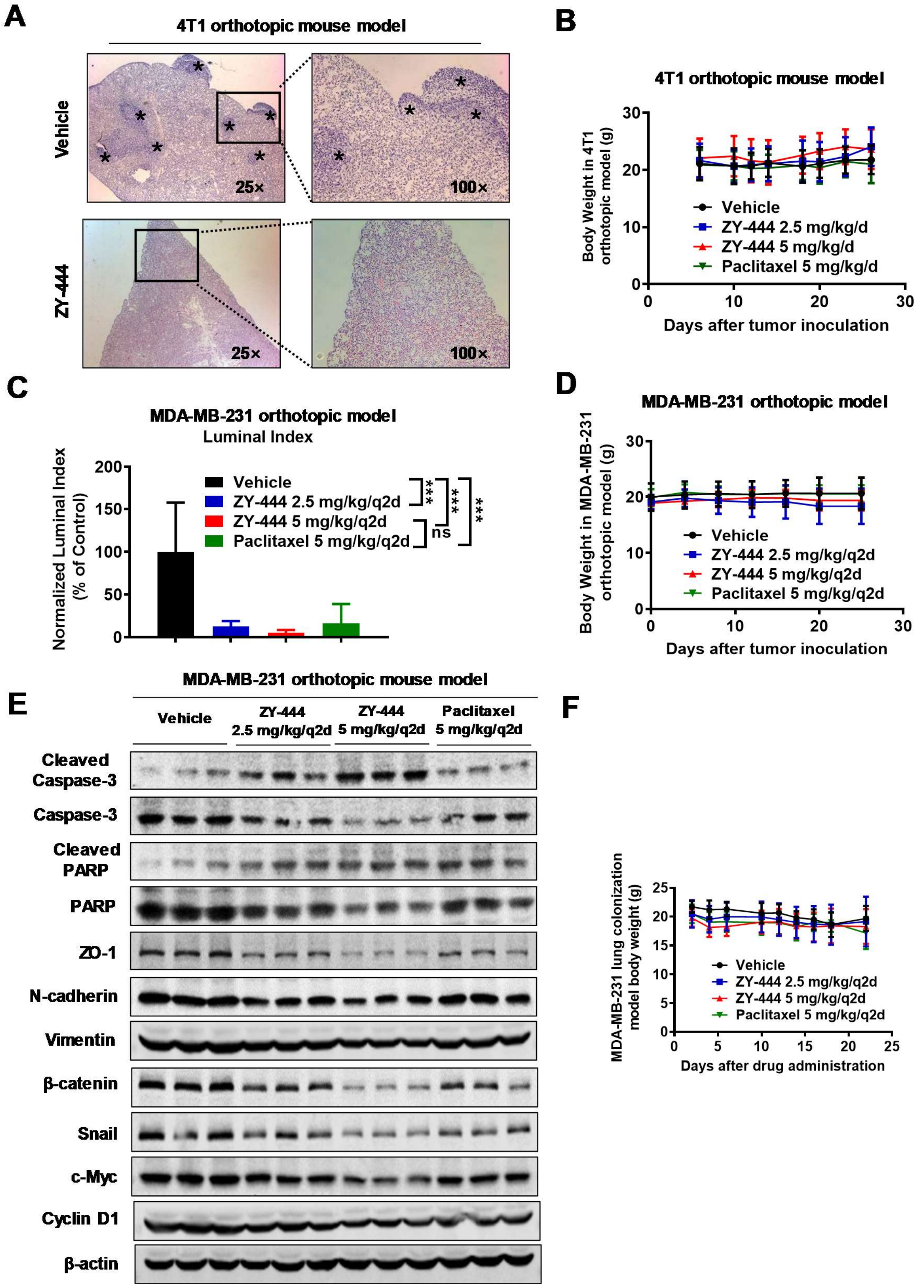

G

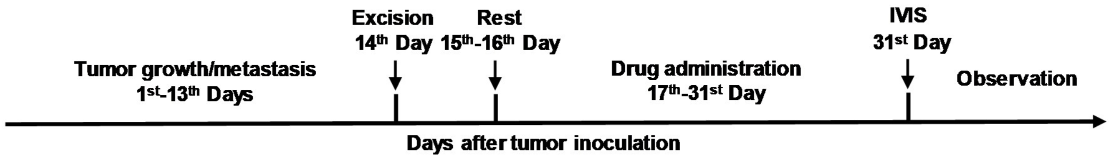
Excision
14th Day
Rest
15th-16th Day
IVIS
31st Day
Tumor growth/metastasis
1st-13th Days
Drug administration
17th-31st Day
Observation
Days after tumor inoculation

H

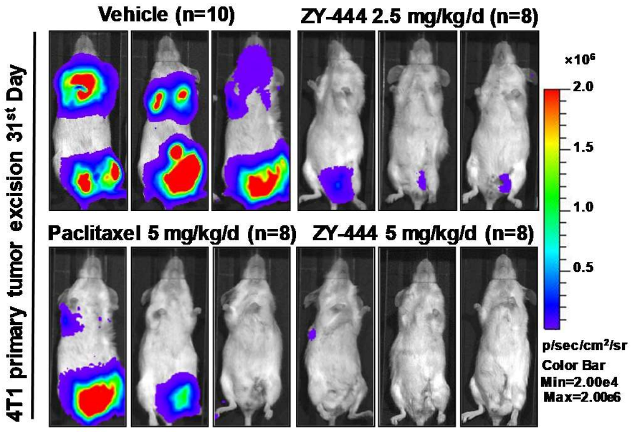
Vehicle (n=10)
ZY-444 2.5 mg/kg/d (n=8)
Paclitaxel 5 mg/kg/d (n=8)
ZY-444 5 mg/kg/d (n=8)
4T1 primary tumor excision 31st Day
×10^6
2.0
1.5
1.0
0.5
p/sec/cm²/sr
Color Bar
Min=2.00e4
Max=2.00e6

I

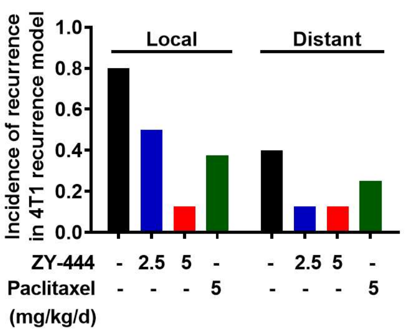
Incidence of recurrence in 4T1 recurrence model
Local
Distant
1.0
0.8
0.6
0.4
0.2
0.0
ZY-444 - 2.5 5 - - 2.5 5 -
Paclitaxel - - - 5 - - - 5
(mg/kg/d)

J

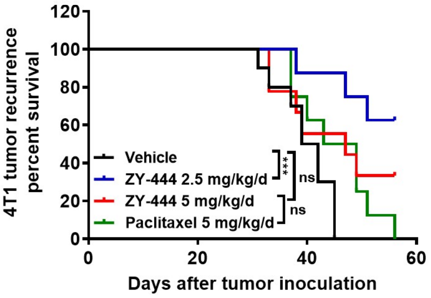
4T1 tumor recurrence percent survival
120
100
80
60
40
20
0
0
20
40
60
Days after tumor inoculation
Vehicle
ZY-444 2.5 mg/kg/d
ZY-444 5 mg/kg/d
Paclitaxel 5 mg/kg/d
ns
ns

Figure S3. ZY-444 inhibits tumor growth and metastasis in vivo.

A) Hematoxylin-eosin staining of lung sections from the 4T1 orthotopic models. Asterisks indicate the tumor nodules in the lungs.

B) Body weight records over 21 days of treatment in the 4T1 orthotopic model.

C) Normalized luminal index measurements over 26 days after tumor implantation in the MDA-MB-231 orthotopic model.

D) Body weight measurements over 26 days of treatment in the MDA-MB-231 orthotopic model.

E) Expression of proteins associated with apoptosis, EMT and Wnt signaling in MDA-MB-231 orthotopic tumors in different treatment groups.

F) Body weight measurements of the MDA-MB-231 orthotopic model.

G) Study design and timeline of the 4T1 tumor recurrence model (n = 8-10).

H) Representative bioluminescent images of tumor excised mice after 14 days' administration of indicated agents.

I) The incidence of local and distant recurrence in the 4T1 recurrence model.

J) Kaplan survival plot of the 4T1 recurrence model.

Data shown are mean ± s.d. $p < 0.001$, ***; ns, not significant.

Lin *et al.* Figure S4

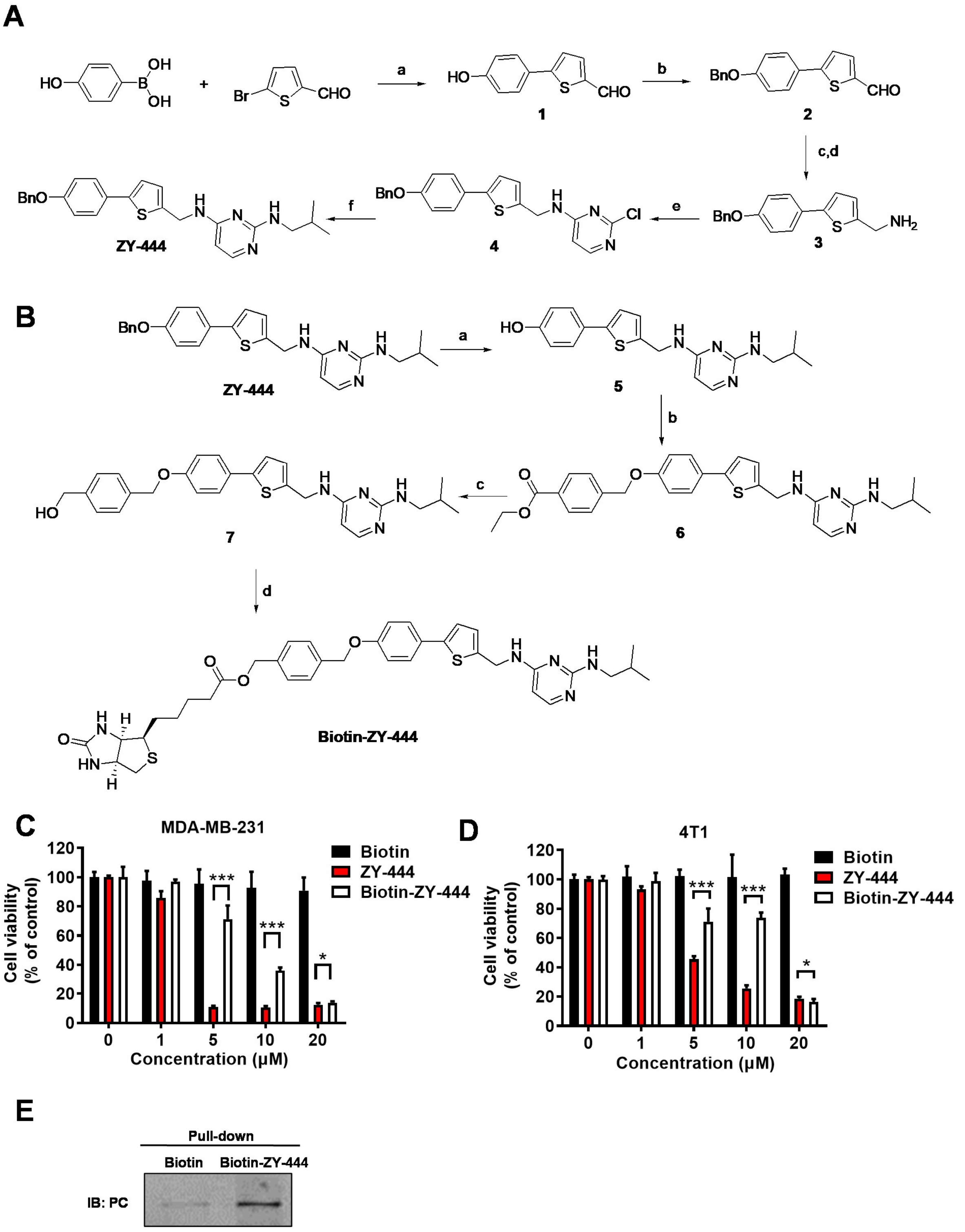

Figure S4. Processes of ZY-444 (A) and biotin-ZY-444 (B) chemical synthesis.

A) Sequential processes of ZY-444 synthesis. Reagents and conditions: (a) $Pd(PPh_3)_4$, Na_2CO_3, EtOH, DME, 90 °C; (b) Benzyl bromide, K_2CO_3, DMF; (c) H_2NOH-HCl, Pyridine, EtOH, 80 °C; (d) Zn, AcOH; (e) 2,4-dichloropyrimidine, DIEA, EtOH, 40 °C; (f) Isobutylamine, DIEA, n-BuOH, 120 °C.

B) Synthetic processes of biotin-ZY-444. Reagents and conditions: (a) $Pd/C/H_2$, MeOH (b) ethyl 4-(bromomethyl)benzoate, K_2CO_3, DMF; (c) $LiAlH_4$, Et_2O, 0 °C ; (d) DCC, DMAP, DCM.

C-D) Effects of biotin, ZY-444 and biotin-ZY-444 on cell viability of MDA-MB-231 (C) and 4T1 cells (D).

E) Antibody against PC was used to detect the presence of precipitated PC in a biotin-assisted pulldown assay.

Data shown are mean ± s.d. $p < 0.05$, *; $p < 0.001$, ***.

Lin *et al.* Figure S5

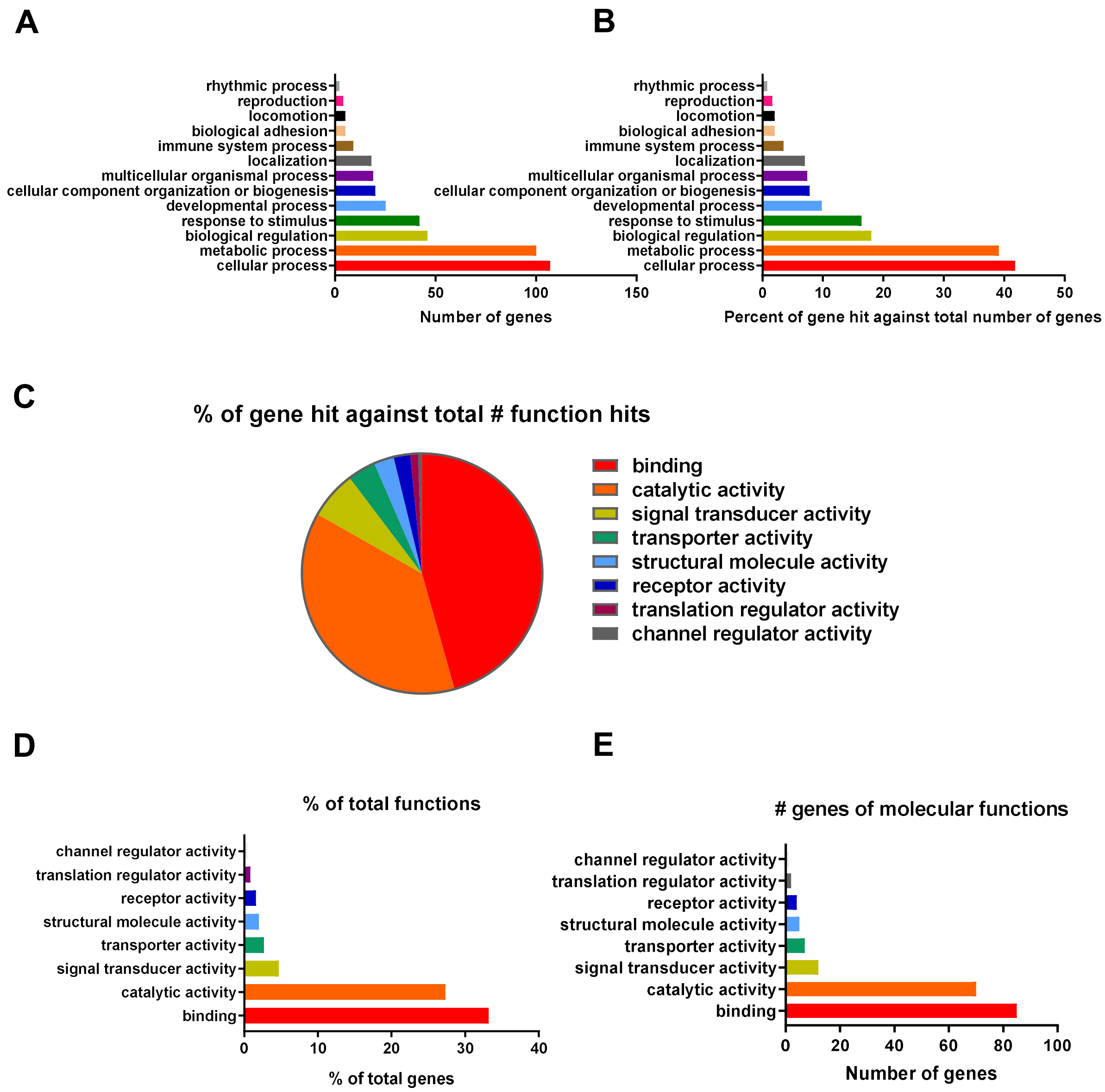

Figure S5. Microarray analysis of ZY-444 treatment in MDA-MD-231 cells.

PANTHER analysis of gene expression profiling on biological processes (A-B) and molecular functions (C-E) in MDA-MB-231 cells treated with vehicle or 5 μM ZY-444.

Lin *et al.* Figure S6

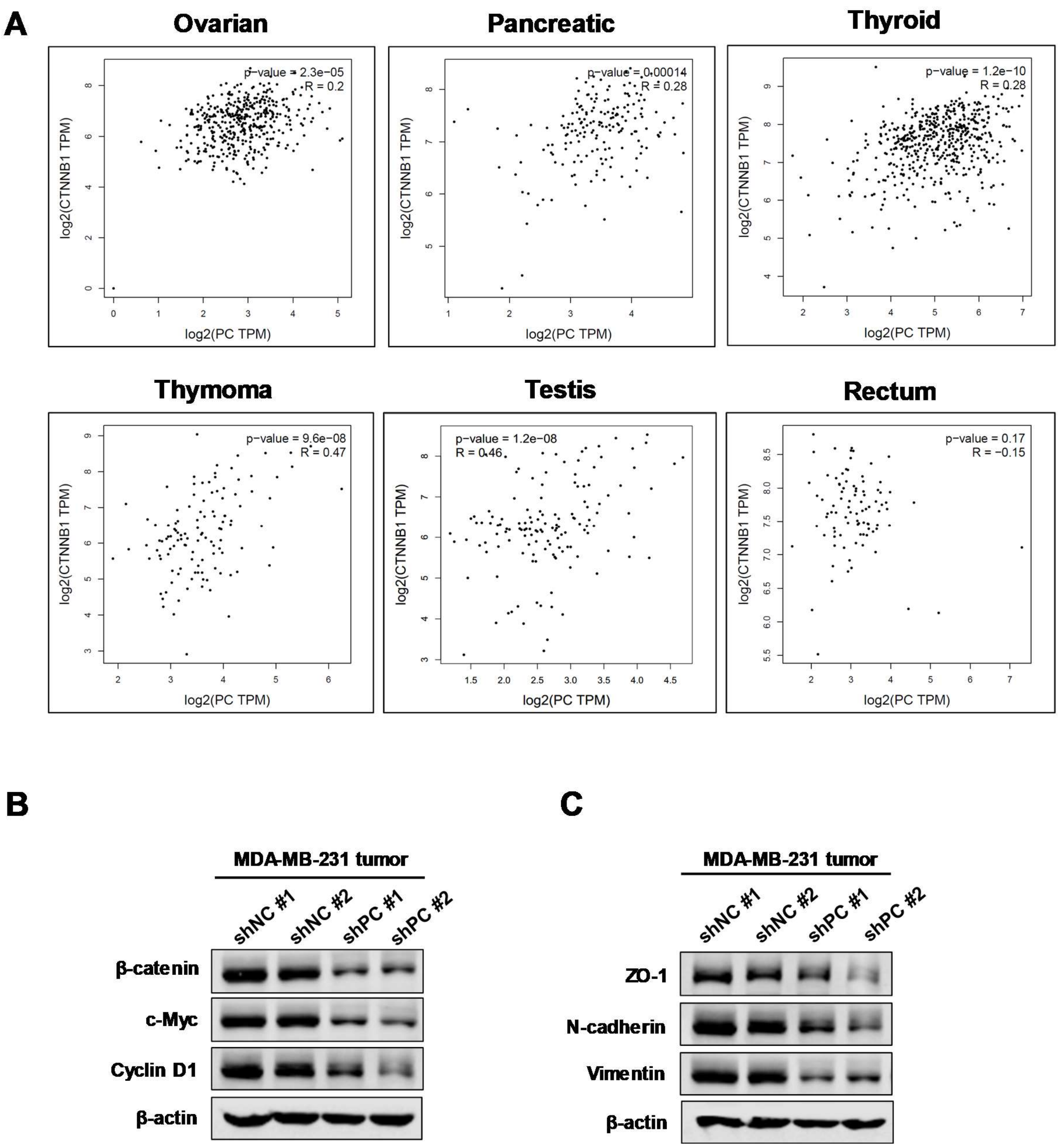

D

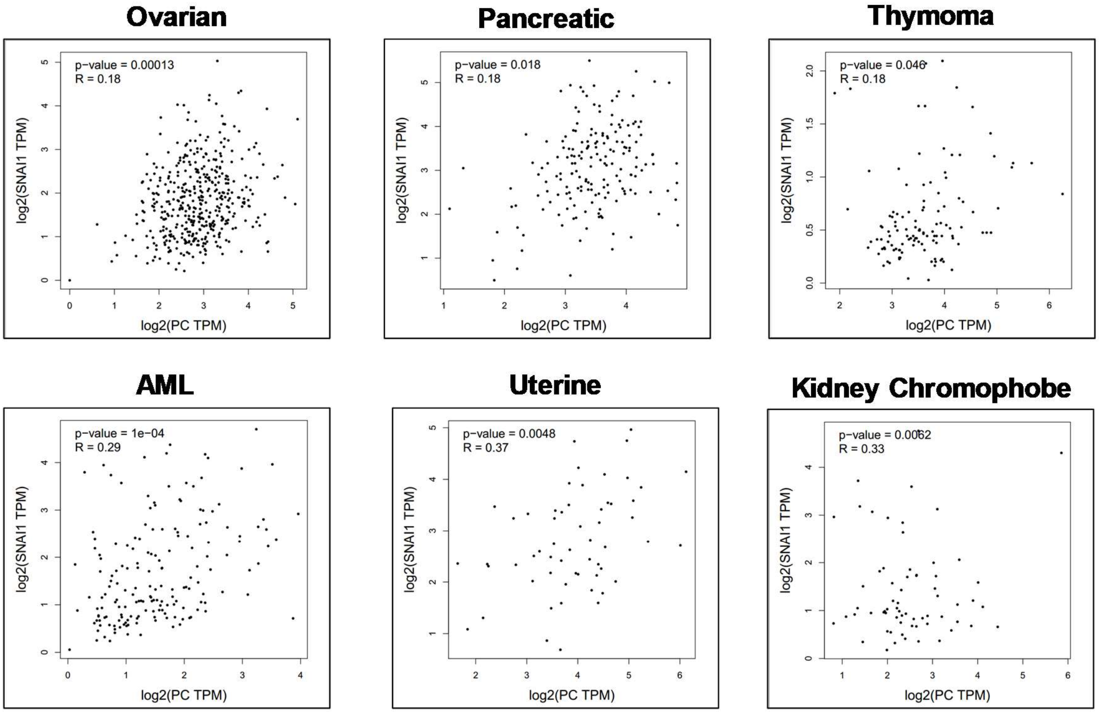
Ovarian
p−value = 0.00013
R = 0.18
log2(SNAI1 TPM)
log2(PC TPM)
Pancreatic
p−value = 0.018
R = 0.18
log2(SNAI1 TPM)
log2(PC TPM)
Thymoma
p−value = 0.046
R = 0.18
log2(SNAI1 TPM)
log2(PC TPM)
AML
p−value = 1e−04
R = 0.29
log2(SNAI1 TPM)
log2(PC TPM)
Uterine
p−value = 0.0048
R = 0.37
log2(SNAI1 TPM)
log2(PC TPM)
Kidney Chromophobe
p−value = 0.0062
R = 0.33
log2(SNAI1 TPM)
log2(PC TPM)

E

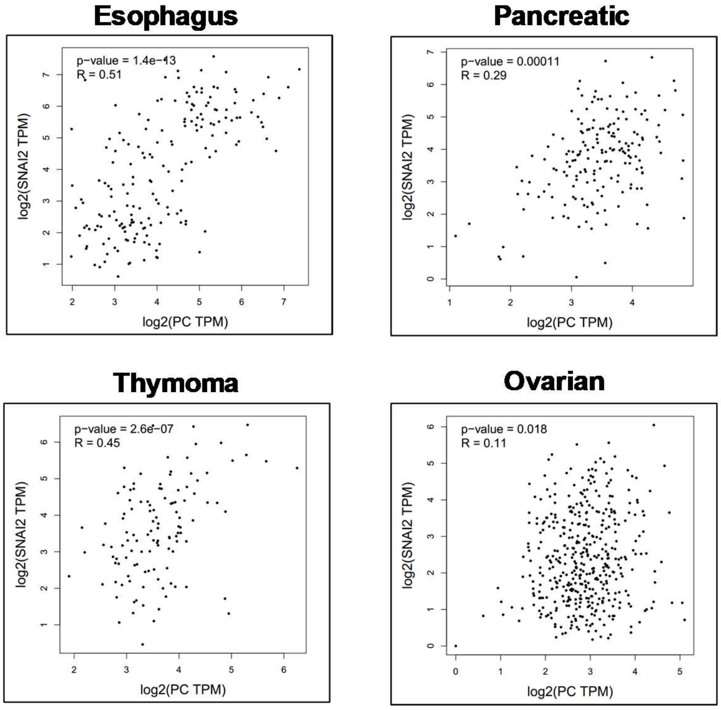
Esophagus
p−value = 1.4e−13
R = 0.51
log2(SNAI2 TPM)
log2(PC TPM)
Pancreatic
p−value = 0.00011
R = 0.29
log2(SNAI2 TPM)
log2(PC TPM)
Thymoma
p−value = 2.6e−07
R = 0.45
log2(SNAI2 TPM)
log2(PC TPM)
Ovarian
p−value = 0.018
R = 0.11
log2(SNAI2 TPM)
log2(PC TPM)

Lin *et al.* Figure S6

F

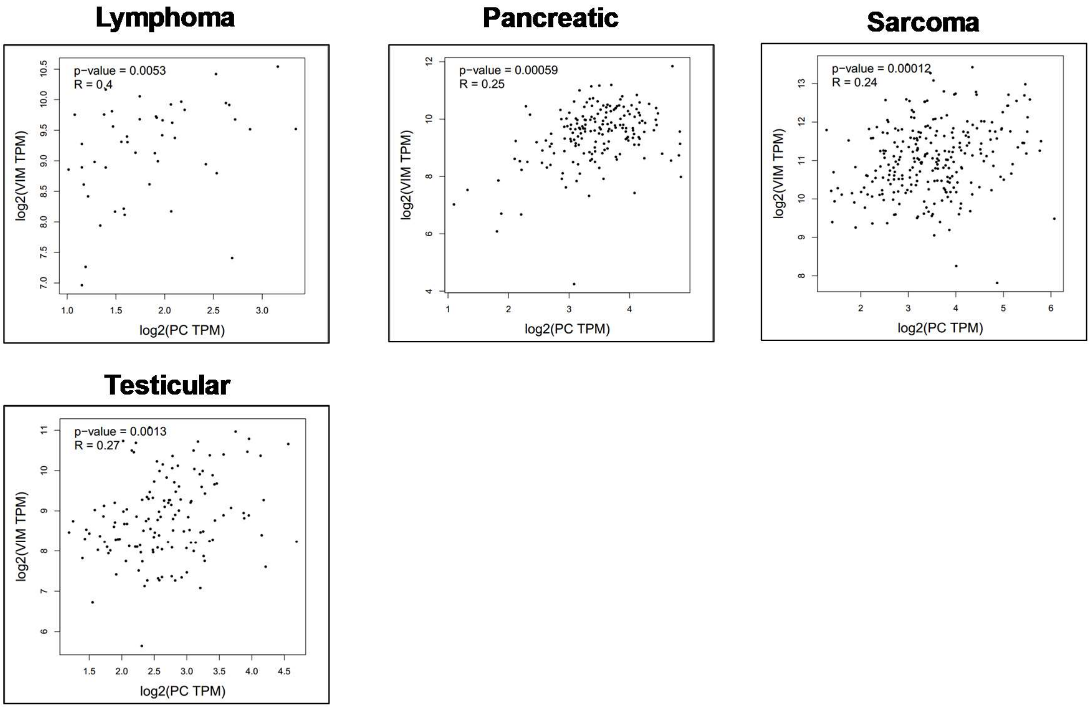

G

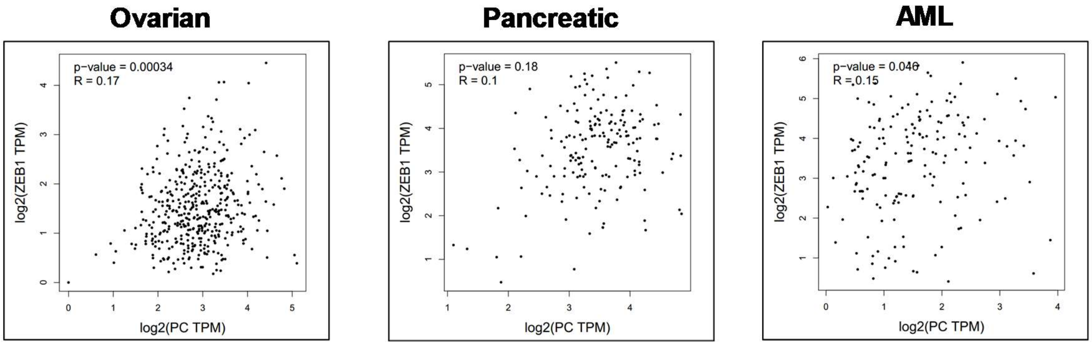

Figure S6. PC is associated with the Wnt/EMT signaling pathway.

A) Co-expression status of PC and β-catenin (CTNNB1) expression in patients with different types of cancer. P-values and correlation coefficient (R) of linear co-expression were calculated by the GEPIA database.

B-C) Expression changes of proteins associated with Wnt signaling (A), EMT (B) and apoptosis (C) in subcutaneous shNC and shPC tumors.

D-G) Co-expression status of PC and Snail (D), Slug (E), Vimentin (F) and Zeb1 (G) expression in patients with different types of cancer. P-values and correlation coefficient (R) of linear co-expression are calculated by GEPIA database.

Lin *et al.* Figure S7

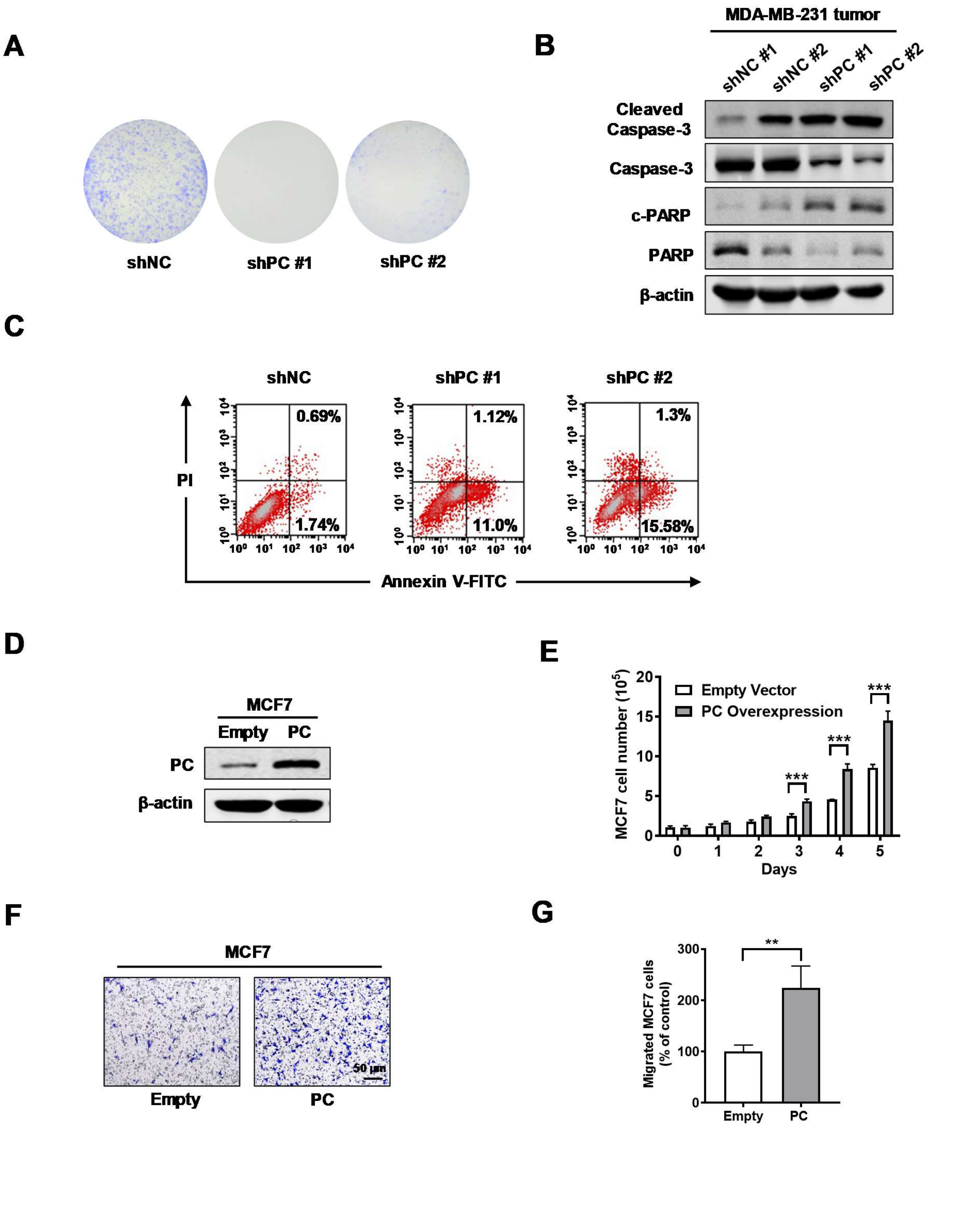

Figure S7. Effects of PC knockdown and overexpression in BCA.

A) Representative images of colony formation for shNC and two shPC expressing cells.

B) Changes in expression of proteins associated with apoptosis in shPC cells.

C) The effects of PC downregulation on apoptosis in shNC and shPC cells.

D-E) The expression of PC in MCF7 cells transduced with empty vector control or PC overexpression vector (D). The cell numbers of MCF7 cells were counted (E).

F-G) MCF7 cells were transduced with either empty or PC overexpression vector (F). The invaded cells were counted and normalized (G).

Data shown are mean ± s.d. $p < 0.01$, **; $p < 0.001$, ***.

Lin *et al.* Figure S8

A

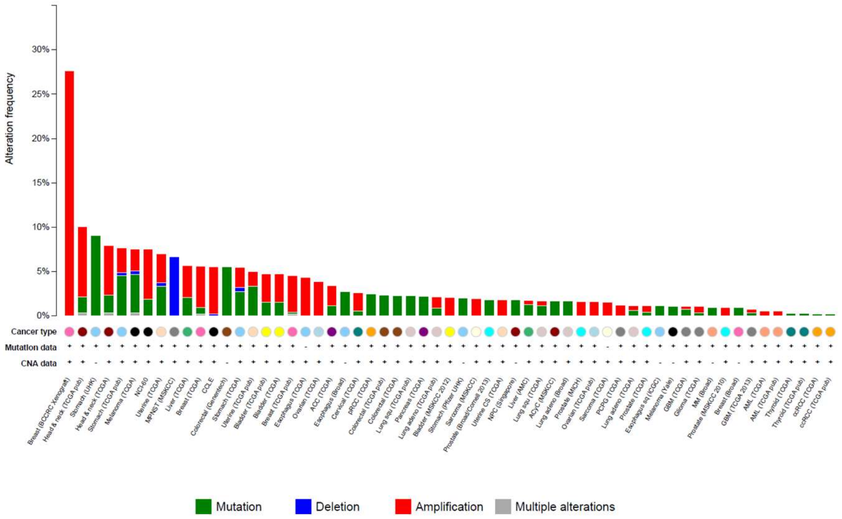

B

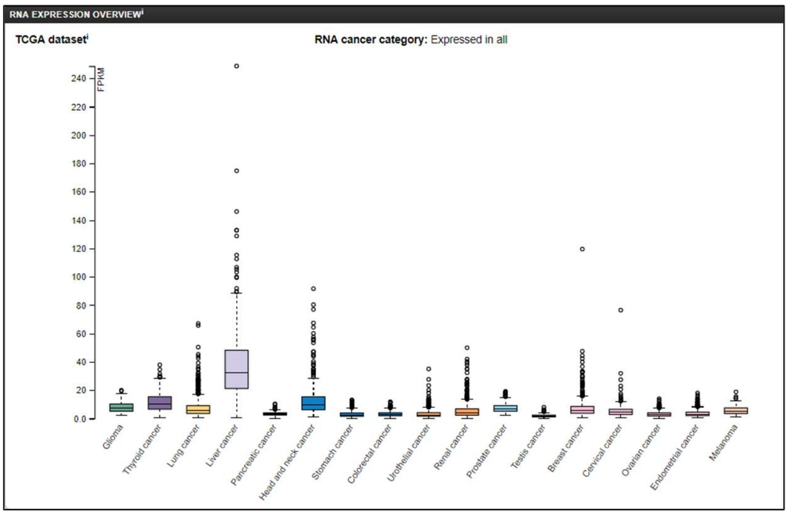

C

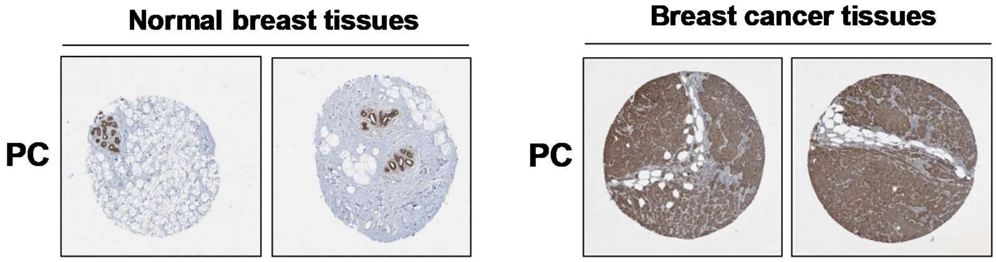

D

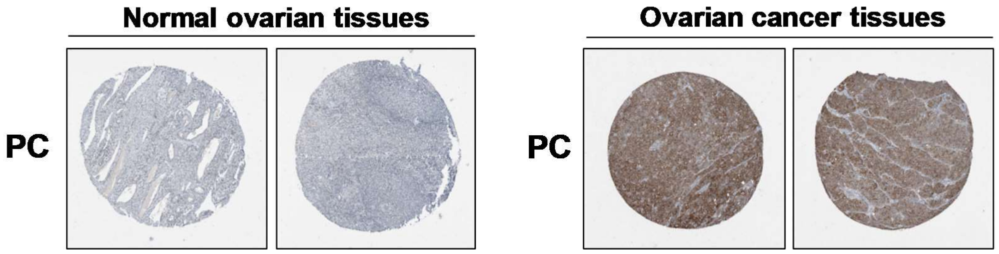

Figure S8. Expression of PC in multiple cancer types.

A) Frequency of PC alterations among different cancer types in TCGA database cancer patients, including mutation (green), deletion (blue), amplification (red) and multiple alterations (grey).

B) Comparison of PC expression in multiple cancer types according to TCGA and THPA databases.

C) Comparison of PC expression in normal human breast tissues *vs.* breast cancer tissues.

D) Comparison of PC expression in normal human ovarian tissues *vs.* ovarian cancer tissues.

3. Supplementary Table Legends

Table S1. Synthesis and Screening of potent anti-metabolism compounds against cancer.

Synthesis of ZY-444 from lead compound WB-339B. The aryl-heteroaromatic groups were highlighted in blue. The series of compounds with aryl-heteroaromatic groups were identified as modulators of mitochondrial respiration at 10 μM and 20 μM (Table S1-a). Negative compounds lacking an aryl-heteroaromatic group did not greatly alter mitochondrial respiration at the same concentrations. "+" and "-" symbols indicated the presence and absence of aromatic-heteroaromatic groups structurally or resulting capability of modulating mitochondrial respiration (Table S1-b).

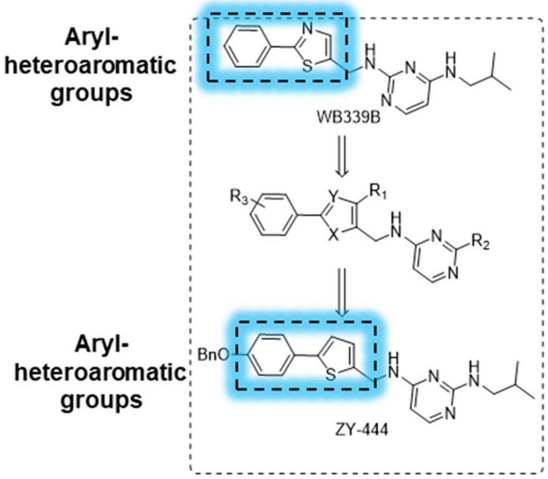

Table S1-a

Compounds	X	Y	R_1	R_2	R_3	Incorporation of aryl-heteroaromatic groups	Inhibition of mitochondria respiration
WB339B	S	N	H	i-Bu-NH-	H	+	+
ZY-325	S	N	CH_3	Et-NH-	H	+	+
ZY-339C	S	N	CH_3	n-Pr-NH-	H	+	+
ZY-353F	S	N	CH_3	n-Bu-NH-	H	+	+
ZY-353D	S	N	CH_3	i-Bu-NH-	H	+	+
ZY-387D	S	N	CH_3	i-Bu-NH-	2-Cl	+	+
ZY-387B	S	N	CH_3	i-Bu-NH-	3-Cl	+	+
ZY-387C	S	N	CH_3	i-Bu-NH-	4-Cl	+	+
ZY-371	S	N	CH_3	i-Bu-NH-	4-F	+	-
ZY-432	S	N	CH_3	i-Bu-NH-	4-Br	+	+
ZY-459	S	N	CH_3	i-Bu-NH-	4-OBn	+	+
ZY-444	S	H	H	i-Bu-NH-	4-OBn	+	+

Table S1-b

Compounds	Incorporation of aryl-heteroaromatic groups	Inhibition of mitochondria respiration
WK-325	-	-
WK-464	-	-
WK-400B	-	-

Table S2. A list of bound proteins of biotin-ZY-444 using mass spectrometry analysis.

Table S2

Accession	Score	Protein ID	Protein Name
P11498	609.06	PC	Pyruvate carboxylase, mitochondrial OS=Homo sapiens GN=PC PE=1 SV=2
Q99613	339.63	EIF3C	Eukaryotic translation initiation factor 3 subunit C OS=Homo sapiens GN=EIF3C PE=1 SV=1
O00203	319.00	AP3B1	AP-3 complex subunit beta-1 OS=Homo sapiens GN=AP3B1 PE=1 SV=3
O43847	236.49	NRD1	Nardilysin OS=Homo sapiens GN=NRD1 PE=1 SV=2
Q70E73	232.88	RAPH1	Ras-associated and pleckstrin homology domains-containing protein 1 OS=Homo sapiens GN=RAPH1 PE=1 SV=3
O60841	229.48	EIF5B	Eukaryotic translation initiation factor 5B OS=Homo sapiens GN=EIF5B PE=1 SV=4
Q14157	203.03	UBAP2L	Ubiquitin-associated protein 2-like OS=Homo sapiens GN=UBAP2L PE=1 SV=2
P55060	194.51	CSE1L	Exportin-2 OS=Homo sapiens GN=CSE1L PE=1 SV=3
Q9H4L5	181.89	OSBPL3	Oxysterol-binding protein-related protein 3 OS=Homo sapiens GN=OSBPL3 PE=1 SV=1
Q13435	179.02	SF3B2	Splicing factor 3B subunit 2 OS=Homo sapiens GN=SF3B2 PE=1 SV=2
P12814	167.43	ACTN1	Alpha-actinin-1 OS=Homo sapiens GN=ACTN1 PE=1 SV=2
O95373	146.24	IPO7	Importin-7 OS=Homo sapiens GN=IPO7 PE=1 SV=1
Q14697	140.51	GANAB	Neutral alpha-glucosidase AB OS=Homo sapiens GN=GANAB PE=1 SV=3
Q9Y2W1	133.25	THRAP3	Thyroid hormone receptor-associated protein 3 OS=Homo sapiens GN=THRAP3 PE=1 SV=2
Q13200	130.25	PSMD2	26S proteasome non-ATPase regulatory subunit 2 OS=Homo sapiens GN=PSMD2 PE=1 SV=3
O14974	129.26	PPP1R12A	Protein phosphatase 1 regulatory subunit 12A OS=Homo sapiens GN=PPP1R12A PE=1 SV=1
Q7Z2W4	127.70	ZC3HAV1	Zinc finger CCCH-type antiviral protein 1 OS=Homo sapiens GN=ZC3HAV1 PE=1 SV=3
P53618	118.53	COPB1	Coatomer subunit beta OS=Homo sapiens GN=COPB1 PE=1 SV=3
Q99459	115.73	CDC5L	Cell division cycle 5-like protein OS=Homo sapiens GN=CDC5L PE=1 SV=2
Q12906	115.55	ILF3	Interleukin enhancer-binding factor 3 OS=Homo sapiens GN=ILF3 PE=1 SV=3

**Q86XP3**	**112.69**	**DDX42**	**ATP-dependent RNA helicase DDX42 OS=Homo sapiens GN=DDX42 PE=1 SV=1**
**Q86W92**	**105.85**	**PPFIBP1**	**Liprin-beta-1 OS=Homo sapiens GN=PPFIBP1 PE=1 SV=2**
**P11586**	**104.95**	**MTHFD1**	**C-1-tetrahydrofolate synthase, cytoplasmic OS=Homo sapiens GN=MTHFD1 PE=1 SV=3**

Table S3. Key binding parameters between ZY-444 and a range of concentrations of PC.

Table S3

Parameter (Unit)	k_a ($M^{-1}\cdot s^{-1}$)	k_d (s^{-1})	K_d (M)	R_{max} (RU)
	1.04E+06	8.42E-4	8.11E-10	74.42

Table S4. PC expression for tumor selectivity in human tissues.

A summary of PC expression in multiple types of tissues and tumors to aid potential patient selection for developing clinical application guidelines. The symbol "+" indicates a higher tumor selectivity in tumor tissues relative to normal tissues. The symbol "-" indicates no selectivity of PC expression present in patients. The symbol "X" indicates higher expression of PC in normal tissues compared to cancer tissues.

Table S4

Organ	PC expression frequency in cancer patient (%)	PC expression score in normal tissues	PC expression for tumor selectivity
Breast	High	Low	++
Thyroid	High	Low	++
Ovary	High	None	+++
Lung	Medium	Low	+
Lymph	Low	None	+
Head and neck	High	Not available	Not available
Urothelial	High	High	-
Liver	High	High	-
Colon	High	High	-
Stomach	High	High	-
Pancreas	High	Medium	+
Endometrial	High	Medium	+
Cervix	High	High	-
Brain	Medium	High or Medium	-
Testis	Low	High	X
Skin	Low	High	X

Kidney	Low	High	X

Table S5. A summary of 5-year survival rates and statistics of log-rank P values in several types of cancer.

Table S5

Cancer type	5-year survival		Log-rank p value
	High	Low	
Ovarian cancer	18%	39%	7.86e-5
Lung squamous cell carcinoma	44%	55%	5.75e-2
Stomach cancer	28%	39%	8.58e-3
Rectum adenocarcinoma	30%	63%	3.78e-2
Endometrial cancer	68%	85%	8.24e-3

Table S6. Summary of cell lines in respective cancer categories.

Table S6

Cancer type	Cell lines	Source
Human breast cancer	MDA-MB-231	ATCC
	MDA-MB-231-luc	Caliper Life Sciences, Inc (Hopkinton, MA)
	MCF7	ATCC
	BT474	
	T47D	
	Hs578T	Chinese Academy of Sciences Committee Type Culture Collection Cell Bank (Shanghai, China)
	Bcap-37	
	BT-549	
Mouse breast cancer	4T1	ATCC
	4T1-luc	Modified from ATCC
Human lung cancer	A549	ATCC
	PC-9	A gift from Dr. Ji (Chinese Academy of Sciences, Shanghai, China P.R)
Human colon caner	HT-29	ATCC
	HCT116	
Human ovarian cancer	ES-2	ATCC
	SKOV3	

	A2780	
Human stomach cancer	MKN45	China Center for Type Culture Collection (Shanghai, China)
	MGC-803	
	BGC-823	
Human liver cancer	SK-HEP-1	ATCC
	SMMC77221	Chinese Academy of Sciences Committee Type Culture Collection Cell Bank (Shanghai, China)
	Huh-7	

Normal tissue type	Cell lines	Source
Human breast epithelial cells	MCF10A	ATCC
Human ovarian epithelial cells	IOSE80	Chinese Academy of Sciences Committee Type Culture Collection Cell Bank (Shanghai, China)

Table S7. Summary of antibodies used.

Table S7

Antibody	Company	Catalog No.
β-actin	Sigma (MI, USA)	A5441
β-catenin	BD Transduction Laboratories (USA)	610154
N-Cadherin	Signalway Antibody (MD, USA)	21474-2
Snail	Cell Signaling Technology (MA, USA)	3879
Slug		9585
Vimentin		5741
ZO-1		8193
Caspase-3		9662
Cleaved-Casapase3		9664
PARP		9532
Cleaved-PARP		5625
BCL2		2876
Bax		2772
PC	Abcam (MA, USA)	ab5548-1
cyclin-D		ab134175
c-Myc		ab32072
